# From nanotags to precision biomedicine: SERS-driven progress and innovation in tumor biomarker profiling, dynamic bioimaging, AI-enhanced diagnostics and therapy

**DOI:** 10.7150/thno.126530

**Published:** 2026-01-01

**Authors:** Xuelin Chen, Tenglong Liu, Weiyuan Chen, Zhexuan Lin, Qiaoxin Zhang, Binbin Zhou, Xia Zhou

**Affiliations:** 1 Department of Pharmacy, Shantou University Medical College, Shantou, Guangdong 515041, China.; 2 Department of Clinical Laboratory, The First Affiliated Hospital of Shantou University Medical College, Shantou, Guangdong 515041, China.; 3 Shenzhen Institute of Advanced Electronic Materials, Shenzhen Institute of Advanced Technology, Chinese Academy of Sciences, Shenzhen, Guangdong 518055, China.

**Keywords:** SERS tags, biomarker detection, bioimaging, artificial intelligence-assisted, tumor diagnosis and treatment

## Abstract

Surface-enhanced Raman spectroscopy (SERS) has revolutionized molecular detection by exploiting the localized surface plasmon resonance phenomenon in noble metal nanostructures, achieving signal enhancement factors up to 10^14^. This transformative technology is reshaping biomedical practices through two fundamental mechanisms: electromagnetic field amplification at nanoscale “hot spots” and chemical charge-transfer effects. This review focuses on the cutting-edge applications of SERS technology in biochemical analysis. Starting by dissecting the nuts and bolts of engineered SERS tags: (1) plasmonic nanoparticles serving as enhancers; (2) Raman reporter molecules acting as fingerprint recognition; (3) A protective shell; (4) selective functionalization with targeting biomolecules, and how they were engineered, optimized, and fine-tuned for precision. Specifically, the review zooms in clinical potential of SERS tags: (1) tumor marker detection and *in vitro* diagnosis; (2) bioimaging; (3) tumor treatment; (4) Artificial intelligence-assisted tumor diagnosis and treatment. Finally, we made the forward look for SERS technology in biomedicine, such as multimodal integration, standardized detection protocols, AI-assisted spectral analysis for point-of-care diagnostics, and large-scale clinical applications.

## 1. Introduction

Surface-enhanced Raman spectroscopy (SERS) has been established as one of the most sensitive and cutting-edge analytical methodologies for trace-level detection, enabling unlabeled and highly specific identification of target analytes. Retaining the intrinsic advantages of conventional Raman spectroscopy, SERS significantly surpasses its precursor in sensitivity and specificity, thereby facilitating the precise detection of analytes at ultralow concentrations. Furthermore, as a non-destructive and non-invasive analytical technique, SERS eliminates the necessity for extensive sample pretreatment while delivering critical insights into the physicochemical properties of target molecules. Its broad applicability encompasses diverse analytical targets, ranging from biomolecules of varying sizes and microorganisms to therapeutic agents with high pharmacological potency. Owing to its unparalleled sensitivity and distinctive molecular vibrational fingerprinting capabilities, SERS has garnered extensive adoption across multidisciplinary analytical domains, including but not limited to clinical diagnostics 1, 2, environmental surveillance 3, 4, and food safety assurance 5. Notably, its integration into biomedical research, from *in vitro* detection to *in vivo* imaging, from tumor diagnosis to multimodal therapy 6. This review focuses on the application progress of SERS in these aspects.

### 1.1. SERS enhancement mechanism

SERS is a method that dramatically enhances signals from inherently weak yet structurally rich Raman scattering. Since its discovery in 1974 7, SERS has evolved from an initial observation of signal amplification on rough silver electrodes to a versatile technology now applied in sensing and imaging, single-molecule detection, and ultrahigh vacuum studies 8-12.

**Figure 1:**
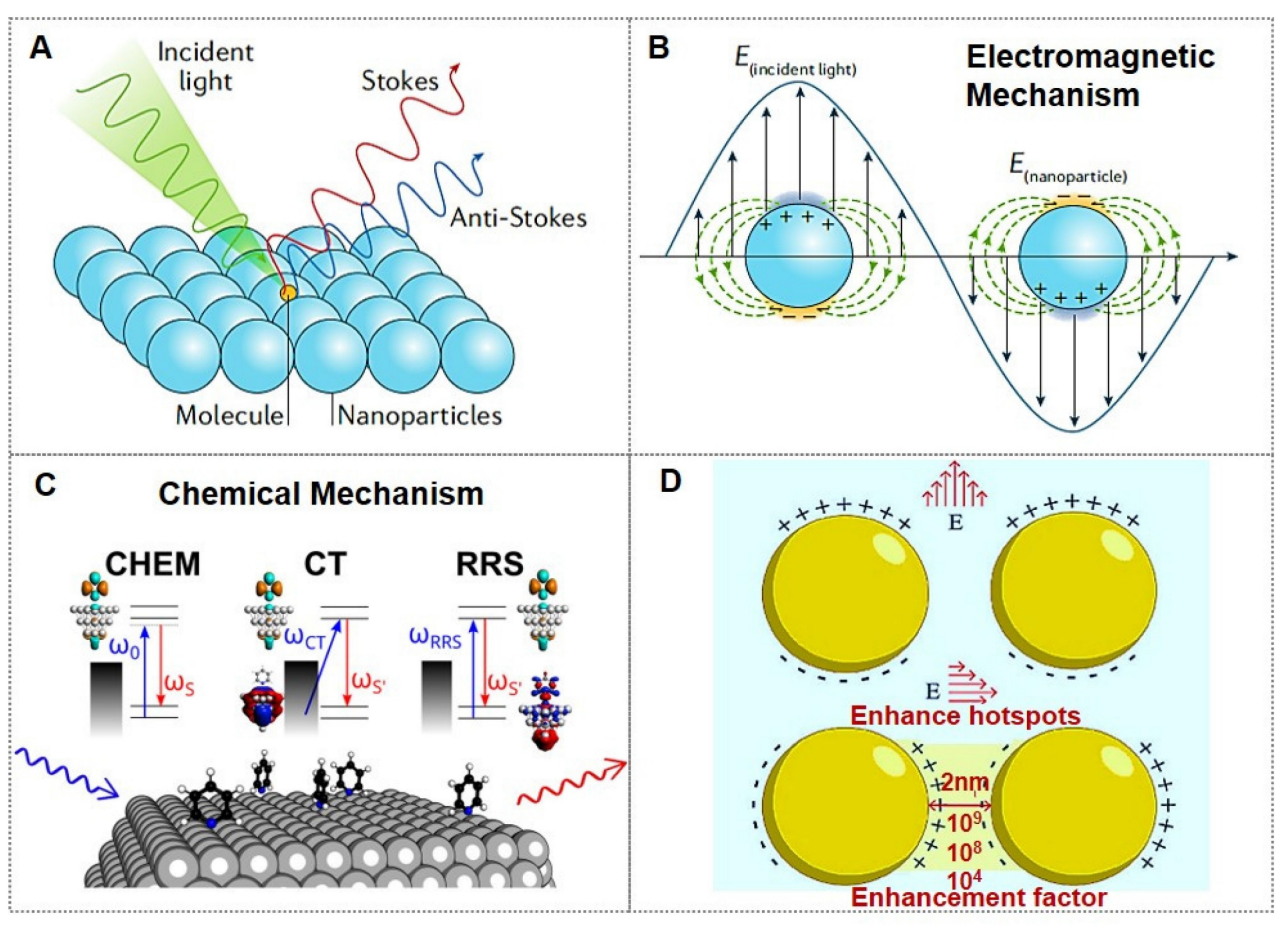
SERS enhancement and mechanism. (A) Schematic illustration of employing precious metal nanoarchitectures for SERS signal amplification. (B) Localized surface plasmon resonance contribution to SERS, electromagnetic enhancement. Reproduced with permission from Ref. 9. Copyright 2022, Springer Nature. (C) Different chemical enhancement mechanisms are illustrated: static chemical mechanism (CHEM), charge-transfer mechanism (CT), and resonance Raman mechanism (RRS). Reproduced with permission from Ref. 11. Copyright 2024, American Chemical Society. (D) Photon-induced plasmon resonance creates localized electromagnetic field enhancements and nanoscale optical hotspot regions. Reproduced with permission from Ref. 10. Copyright 2025, Elsevier.

Owing to its immense enhancement effect, SERS currently enables detection at the single-molecule level (Figure 1A). Various physical and chemical enhancement mechanisms have been proposed to explain the phenomenon, with electromagnetic mechanisms (EM, Figure 1B) and chemical mechanisms (CM, Figure 1C) being the two common mechanisms 8-12. The electromagnetic mechanism is generally considered dominant in SERS enhancement 11, 13, providing an intensity amplification of 4 to 11 orders of magnitude. Although the chemical mechanism contributes a modest enhancement factor of approximately 10^3^, it significantly modifies SERS spectral characteristics. Consequently, the total SERS enhancement factor arises from the synergistic interplay of both electromagnetic and chemical mechanisms. When the gap of the Au nanoparticle dimer decreased from 10 nm to 2 nm, the enhancement factor (EF) increased from 10^4^ to 10^9^, resulting in a “hotspots” effect (Figure 1D) 10. The implementation of a dual-strategy methodology for attaining ultrasensitive SERS detection involves the synergistic integration of an analyte coupling/enrichment strategy with plasmonic hotspot engineering. To achieve highly sensitive SERS applications, the multi-dimensional research progression encompasses three principal innovation pathways: (1) hotspot engineering through different dimensions of nanomaterials; (2) analyte manipulation via chemical coupling strategies, chemical analyte directing strategies, and molecular enrichment strategies; (3) materials hybridization of two-dimensional materials, semiconductors, and stimuli-responsive polymer to achieve ultrasensitive SERS.

### 1.2. The main advantages of SERS application in biomedicine

In recent years, the burgeoning demand for real-time, point-of-care analytical platforms in biomedical sensing has become increasingly pronounced. In clinical scenarios, *in situ* detection of low-molecular-weight analytes, field-deployable forensic analysis, and continuous therapeutic drug monitoring 14.

As an emerging fingerprint recognition technology with ultra-sensitive quantitative capabilities, SERS has demonstrated superior performance in biomedicine primarily due to the following merits: (1) SERS signals reflect intrinsic molecular vibrational fingerprints, enabling multiplex biomarker identification through characteristic spectral signatures; (2) The exponentially enhanced Raman signals permit ultrasensitive detection of trace-level biomarkers in serum; (3) The weak Raman scattering of water molecules minimizes background interference during aqueous-phase biomarker detection; (4) Compatibility with near-infrared lasers facilitates optical fiber transmission, enabling *in situ* biomarker detection in biological systems; (5) Simplified sample preparation and rapid detection procedures, combined with advancements in portable/handheld Raman spectrometers, support online, real-time, and point-of-care diagnostics. These collective advantages position SERS technology as a highly promising approach for tumor biomarker analysis.

Despite progress, significant challenges hinder SERS deployment for practical biomedicine. A key barrier is conducting on-site testing, how to utilize the SERS substrate to efficiently and conveniently extract the sample to be tested on irregular surfaces while ensuring post-sampling stability. Simultaneously, achieving atomic molar sensitivity alongside assay reproducibility remains difficult. Therefore, rationally engineering SERS-active architectures with tailored properties for environmental adaptability, signal fidelity, and robustness is essential for advancing biosensing.

### 1.3. Comparison between SERS and traditional analytical techniques

SERS has demonstrated transformative advantages in the field of biomedical detection and imaging, leveraging its ultra-high sensitivity, multiplexing capability, and potential for *in vivo* applications. It is particularly well-suited for trace-level multi-target analysis and precision intraoperative guidance. However, its clinical translation still requires addressing challenges related to substrate reproducibility and standardization.

Conversely, conventional techniques maintain irreplaceability in domains requiring standardized assays, e.g., Enzyme-Linked Immunosorbent Assay (ELISA) 15 / Real-Time Quantitative PCR (qPCR) 16, dynamic imaging (e.g., fluorescence) 17, and multi-omics analysis (e.g., mass spectrometry) 18. The analysis and comparison of the optimal and limited application scenarios of SERS technology and other traditional analytical techniques are presented in Table 1. Future integration of multiple technologies (e.g., SERS-mass spectrometry integration) will maximize diagnostic capabilities.

This review systematically examines cutting-edge applications of SERS tags in biomedicine. The engineering principles and fabrication methodologies underpinning SERS tags' design were first outlined. Next, their dual functionality as biosensors and imaging probes was critically analyzed: (1) ultrasensitive detection of disease biomarkers in biofluids and intracellular compartments; (2) multimodal imaging spanning *in vitro* cellular tracking to *in vivo* tumor mapping; (3) pioneering clinical innovations are emphasized, particularly intraoperative tumor margin identification and integrated theranostic platforms; (4) by addressing translational challenges, including signal standardization and biocompatibility optimization to bridge laboratory innovation with clinical adoption.

## 2. SERS hotspots engineering

SERS hotspots are subwavelength regions of intense localized electromagnetic fields, typically confined within nanogaps (<10 nm) of noble metal nanoparticles or nanostructured surfaces. Upon laser excitation, plasmonic coupling between adjacent nanoparticles induces collective electron oscillations (surface plasmon polaritons), generating localized electric field enhancements. These spatially confined regions, where molecular adsorbates experience maximal Raman signal amplification, are collectively referred to as “SERS hotspots” 12, 19, 20. The electromagnetic enhancement mechanism dominates SERS activity, contributing up to 10^8^-10^11^ signal amplification factors.

The advent of nanoscale fabrication technologies has empowered the design and synthesis of architecturally varied SERS substrates with hotspots. When designing high-performance SERS substrates, three critical factors must be addressed: (1) Generation of abundant electromagnetic hotspots: sharp-tipped metallic nanostructures (e.g., nanostars and triangular nanoprisms) exhibit stronger near-field plasmonic coupling compared to spherical nanoparticles and nanorods, thereby providing superior SERS enhancement. This phenomenon arises from the intensified localized electromagnetic fields at nanoscale gaps and sharp protrusions. (2) Mitigation of molecular contamination: competitive adsorption of non-target molecules onto electromagnetic hotspots can compromise signal specificity. (3) Precise control over near-field hotspot distribution and reproducibility of SERS signals remains a focal point in substrate optimization. Tailoring the morphology and dimensions of plasmonic materials to fabricate high-performance functionalized SERS-active substrates represents a promising strategy for achieving ultra-sensitive SERS sensors 19, 20.

### 2.1. Hotspots construction in different dimensions

Strategies for SERS hotspots engineering, optimizing focus on: (1) Zero/one-dimensional (0D/1D) plasmonic nanoarchitectures; (2) Two-dimensional (2D) plasmonic metasurfaces; (3) Three-dimensional (3D) hierarchical superlattices. Different application scenarios have different strategies for hotspot construction. We have analyzed and summarized the commonly used application scenarios and construction strategies, as shown in Table 2 21-24.

In summary, the dimensional strategy for constructing SERS hotspots is dictated by specific application requirements (e.g., sensitivity, reproducibility, uniformity, throughput, and sample form).‌ Modern SERS substrate design frequently ‌integrates the advantages of multiple dimensions‌. Examples include assembling 0D particles into arrays on 2D planes, loading 0D/1D nanounits within 3D porous frameworks, or constructing 0D-1D-2D hierarchical heterostructures. This multidimensional integration aims to achieve SERS hotspots with ‌high density, high intensity, high uniformity, and multifunctionality‌.

### 2.2. DNA-nanostructure-enabled SERS hotspots engineering

**Figure 2:**
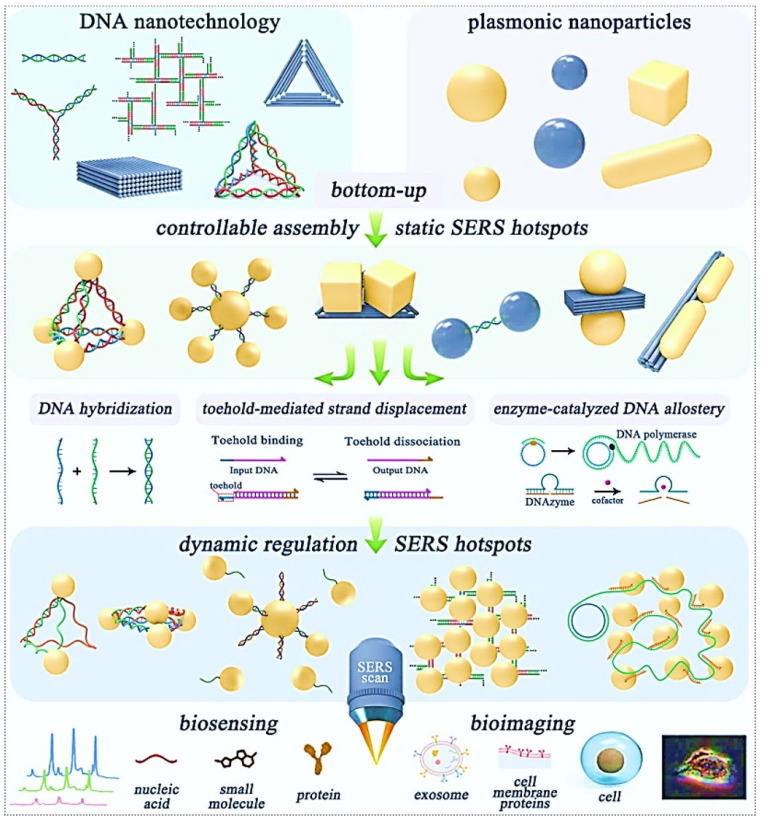
DNA-nanostructure-enabled engineering of SERS hotspots: systematic assembly, dynamic control, and biomedical applications. Reproduced with permission from Ref. 12. Copyright 2025, The Royal Society of Chemistry.

Moreover, DNA-based nanostructures, distinguished by their unparalleled designability and precise programmability, have demonstrated significant potential for the systematic assembly and fine-tuned control of SERS hotspots. While the utilization of DNA nanotechnology in manipulating SERS hotspots has gained considerable attention in recent research, a thorough consolidation of this domain remains absent 25, 26. Recently, a review published by the Wang group investigated established protocols for constructing static SERS hotspots employing DNA frameworks of varying dimensionalities as either connectors or scaffolds. The discussion progresses to address dynamic modulation techniques for SERS hotspots enabled by DNA architectures, including: Topology alterations induced by DNA strand hybridization; Toehold-mediated structural rearrangement (TMSD); Allosteric control via enzymatic DNA manipulation (Figure 2) 12. Recent advancements in DNA-guided hotspot regulation for biological sensing and imaging applications are highlighted.

The DNA-enabled fine-tuned control of SERS hotspots for biological sensing and imaging confronts multifaceted obstacles, yet exhibits significant transformative potential. Through sustained interdisciplinary advancements, these limitations are poised to be systematically addressed, ultimately enabling practical implementations in this emerging research domain.

## 3. Construction of SERS tags

In general, SERS assays are categorized into two approaches: label-free (direct) detection and labeled (indirect) detection 27, 28. The direct label-free SERS assay enables the acquisition of intrinsic Raman spectra from molecules adsorbed directly onto the SERS-active substrate. While direct SERS analysis significantly simplifies the detection protocol, it is highly susceptible to interference from the biomolecular environment and requires both high-efficiency SERS substrates and strong binding interactions between nanoparticles and target molecules. Furthermore, not all analytes can be differentiated based on their intrinsic SERS signatures. In contrast, the indirect SERS assay employs Raman reporters-labeled tags for target detection, allowing diverse analyte types 28. Consequently, the choice of Raman technique, direct or indirect, should be tailored to the specific sample matrix, followed by the rational design of corresponding plasmonic nanostructured substrates or SERS nanotags to optimize detection performance.

**Figure 3:**
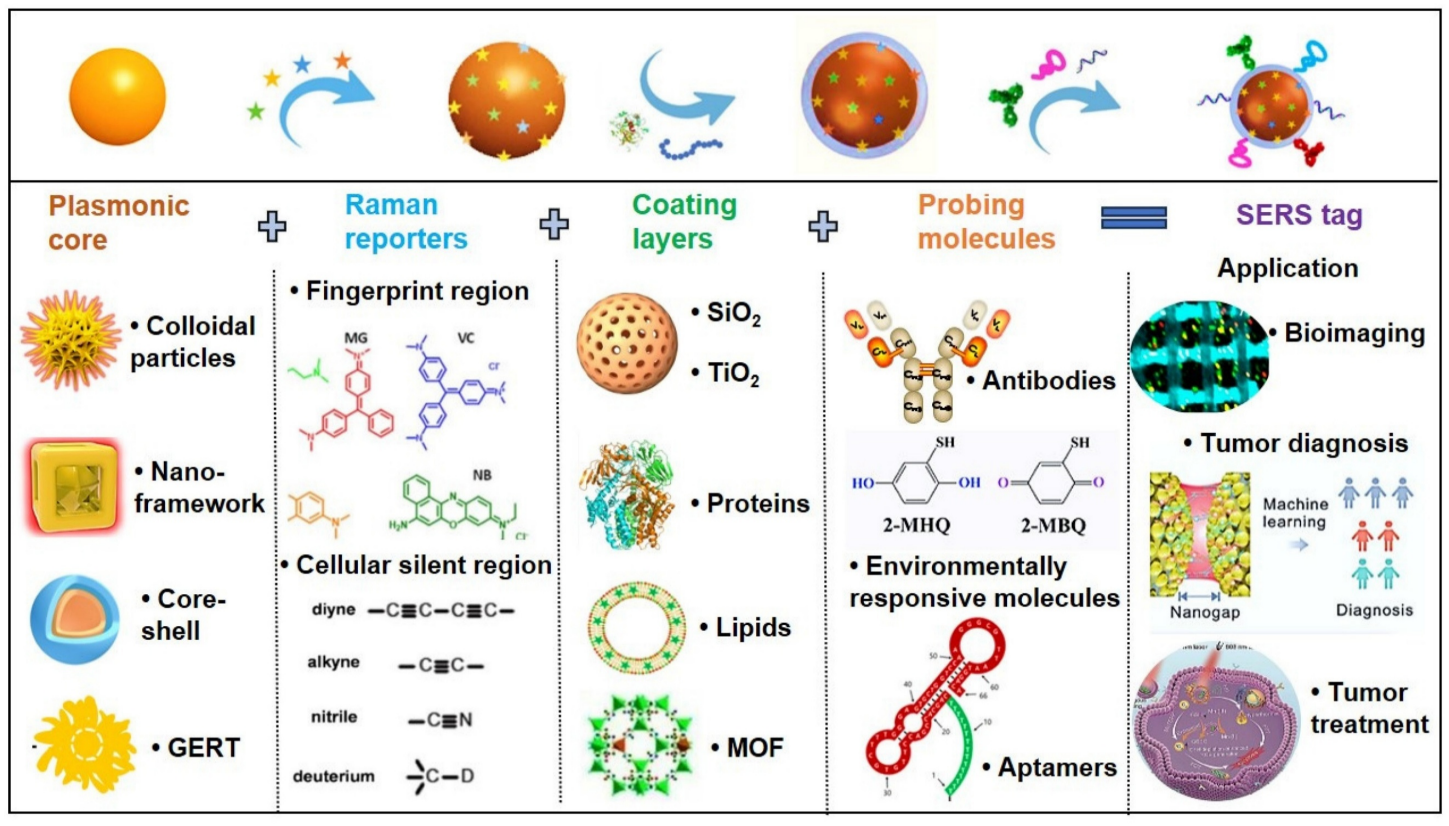
Architectural decomposition of core-configurable SERS-active tags delineates four functional components: (1) Plasmonic core; (2) RaR molecules; (3) Stability coating; (4) Probing molecules for biorecognition.

To achieve indirect SERS detection based on antigen-antibody (or other bioreceptor) interactions, SERS tags are indispensable. SERS tags are hierarchically structured with four functional components: (I) plasmonic core serving as enhancers; (II) Raman reporter molecules (RaRs) acting as fingerprint tags; (III) a protective shell or coating layer; (IV) selective functionalization with probing biomolecules (Figure 3) 29, 30. Next, we will provide detailed explanations for each of the four components of the SERS tags.

### 3.1. Plasmonic core

This review will focus on three primary plasmonic core categories: (1) metal colloidal substrate; (2) metal nano-framework substrates; (3) core-shell nanostructure.

#### 3.1.1. Metal colloidal nanoparticles

Owing to their simplicity of fabrication and low cost, metallic nanoparticle colloids have been the most widely utilized SERS-active substrates in early-stage research. Under visible and near-infrared light excitation, gold (Au), silver (Ag), and copper (Cu) exhibit exceptional performance as SERS substrates, with Au and Ag nanoparticles (NPs) being preferentially adopted due to their superior plasmonic enhancement capabilities. For Au and Ag NPs, the LSPR peaks typically occur at approximately 520 nm and 400 nm, respectively. These colloidal substrates are predominantly synthesized via chemical reduction methods, where the size, morphology, and surrounding dielectric environment of the nanoparticles critically influence their plasmonic resonance properties. Consequently, achieving high SERS enhancement necessitates the selection of NPs with optimized geometries (e.g., spherical, anisotropic) and controlled dimensions to enable dense packing of near-field hotspots. The correlation between nanoparticle size and SERS efficiency has been extensively investigated by numerous research groups 31, highlighting the importance of precise nanoscale engineering for maximizing electromagnetic field amplification. For instance, Ming Li *et al.* 32 reported a class of porous nanostructures comprising cubic Au-Ag alloy nanoframes (pc-AuAg NSs) integrated with abundant nanopores.

The nanogaps and sharp edges/tips in nanostructures significantly amplify Raman scattering intensity, with the EF increasing proportionally to the sharpness of the taper or tip and the number of protrusions. Studies demonstrate that near-field interactions and hotspot formation are pivotal for achieving strong SERS signals. Beyond the intrinsic morphology and size of nanoparticles, a highly effective strategy for signal amplification involves controlled nanoparticle aggregation. Recent advancements highlight that colloidal nanoparticle aggregation improves SERS reproducibility. For instance, aggregation can be induced by introducing salt ions (e.g., NaCl, NaNO_3_) into the colloidal solution 33. The use of aggregated nanoparticles in liquid-phase analysis represents a practical SERS detection methodology, leveraging easily synthesized or commercially available materials while delivering substantial signal enhancement.

#### 3.1.2. Metal nano-framework structure

Electromagnetic field localization predominantly occurs at nanoscale geometric singularities‌ (e.g., vertex regions or sub-10 nm interparticle junctions) within plasmonic architectures. The optimization of spectrally flat enhancement profiles, coupled with nanometric spatial regulation of plasmonic coupling efficiency and near-field magnitude, constitutes fundamental methodologies for improving both the detection threshold and reproducibility of SERS measurements 34. A breakthrough in this domain was demonstrated by Sungho Park *et al.*, who enabled atomic-level precision in synthesizing discrete nanoparticles, facilitating the creation of monodisperse metallic nanoframes (NF) with programmable optical properties. The technique permits on-demand modulation of both nanoscale geometric parameters and interparticle coupling distances.

**Figure 4:**
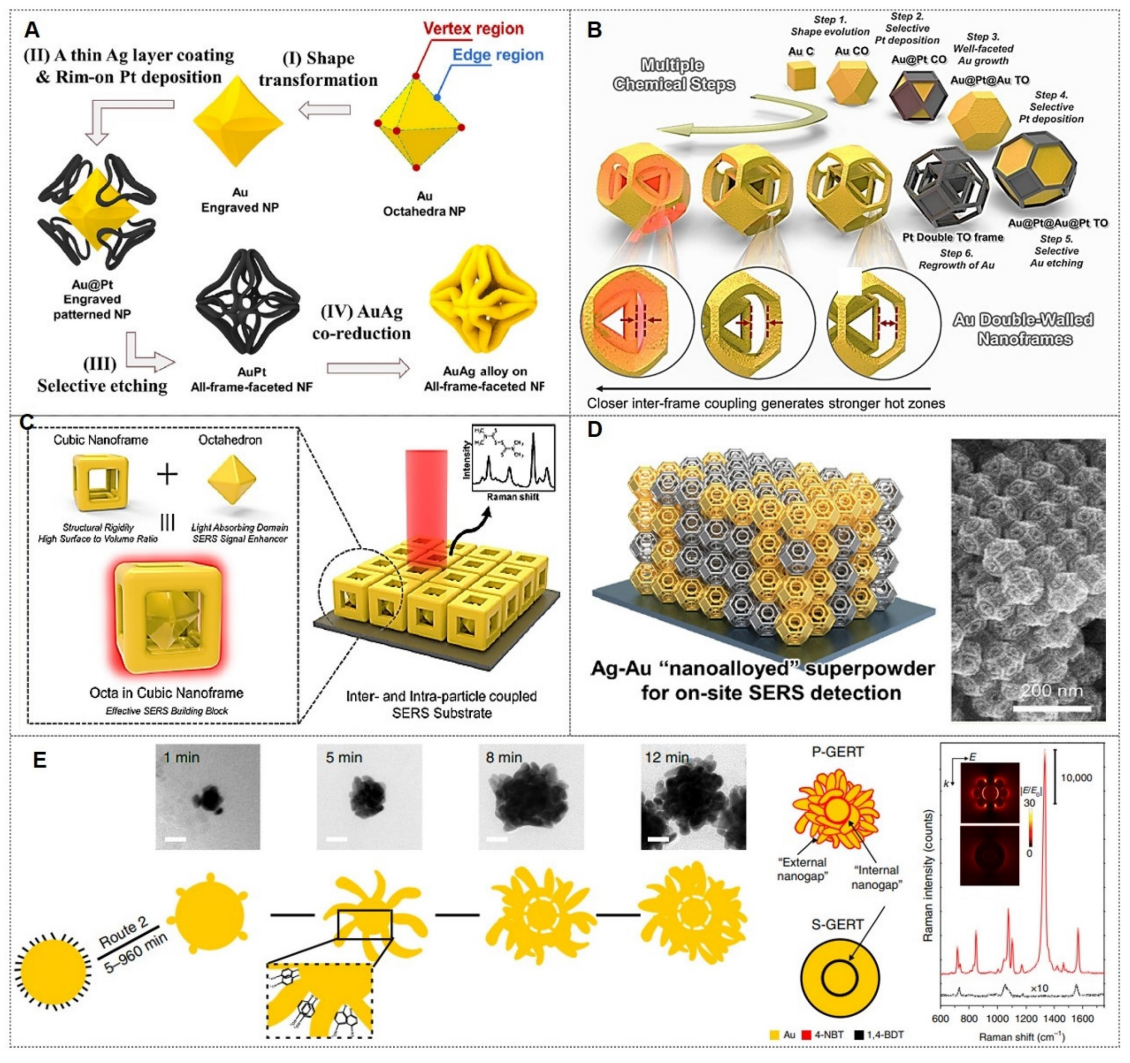
(A) Controlled bottom-up construction of edge-continuous octahedral framework nanostructures‌, accompanied by mechanistic elucidation. Reproduced with permission from Ref. 36. Copyright 2022, American Chemical Society. (B) Multiple stepwise synthetic pathways for Au double-walled nanoframes. Reproduced with permission from Ref. 38. Copyright 2023, American Chemical Society. (C) 3D topological representation‌ of plasmonic octahedral inclusions within cubic framework matrices, and SERS amplification. Reproduced with permission from Ref. 39. Copyright 2024, American Chemical Society. (D) Spatiotemporal mapping of Ag-Au bimetallic phase integration‌ at the nanoscale, highlighting the role of compositionally graded nanoarchitectures in optimizing localized plasmon resonance. Reproduced with permission from Ref. 40. Copyright 2024, American Chemical Society. (E) Schematic diagram of the structure of gap-enhanced Raman tags and their enhanced contrast. Reproduced with permission from Ref. 43. Copyright 2019, Springer Nature.

Their group first reported synthetic strategies for constructing web-above-ring (WAR) and web-above-lens (WAL) nanostructures 35. Within this unified WAL nanostructure, plasmonic hotspots, nanopores, and thermal lensing effects are spatially orchestrated to synergistically amplify SERS signal intensity. Methodological advancement was evidenced through the bottom-up assembly of defect-free octahedral framework nanostructures‌, wherein eight branched plasmonic concentrators with threefold symmetry were spatially organized as integrated electromagnetic field amplifiers, achieving optimal near-field enhancement factors at designated coupling nodes (Figure 4A) 36. Further advancements included a stepwise synthesis protocol for Au triangular nanoframes with inscribed nanorings, exhibiting exceptional structural robustness under high-temperature and oxidative environments 37. The inscribed nanoring configuration induces a "lightning rod effect," enabling single-particle analyte detection via SERS with enhanced sensitivity (Figure 4B) 38. This structurally intricate Au dual-walled nanoframe contains sub-nanometer gaps, demonstrating significantly amplified near-field enhancement for weakly adsorbed SERS analytes, particularly optimized for gas-phase detection.

Additionally, they introduced a unique architecture comprising a solid Au octahedron enveloped by a cubic Au nanoframe, generating internal nanogaps within a single entity 39. The robust core-frame structure enhances near-field focusing, thereby increasing hotspot density. This design achieved ultra-sensitive detection of 2-naphthalenethiol and thiram, showcasing its utility in trace analyte monitoring (Figure 4C). Advancing prior work, the team pioneered a dual-component SERS platform‌ comprising plasmonically coupled Pt@Ag and Pt@Au heterostructures with faceted octahedral morphology. The platinum-based truncated octahedron (TOh) template functions as a structural matrix for epitaxial growth of noble metal (Au/Ag) nanoshells, preserving identical crystallographic orientations across different plasmonic phases while enabling hybridized “nanoalloyed” configurations (Figure 4D) 40. Field validation confirmed these engineered plasmonic meta-materials effectively identified molecular signatures in complex pollutant matrices, underscoring their translational viability for real-world SERS implementations. The comparative analysis of six metal nano-framework structures for SERS detection is shown in Table 3. This analysis provides a theoretical basis for selecting optimal nano-framework structures based on specific application requirements.

#### 3.1.3. Core-shell nanoparticles

Bare nanoparticles may induce host tissue damage and demonstrate inherent cytotoxicity during biological testing. In contrast to bare nanoparticles, core-shell nanoparticles exhibit reduced cytotoxicity, enhanced dispersion stability, superior biocompatibility, and improved chemical stability. Furthermore, nanoengineered core-shell architectures have been demonstrated to generate stronger near-field enhancement effects compared to singular nanoparticle systems. For example, Li's research team‌ delineated advancements in engineered core-shell nanoarchitectures tailored for accelerated SERS screening of agrochemical contaminants, encompassing diverse configurations such as Fe_3_O_4_@metals, SiO_2_@ metals, metals@SiO_2_, metals @metals oxide 41. ‌Concurrently, Sotiriou and collaborators‌ established an industrially viable combustion-derived deposition protocol for fabricating homogeneous SERS-active substrates, wherein thermally generated silver nanoparticle clusters self-organize into spatially controlled plasmonic arrays on solid supports, concurrently augmenting electromagnetic field localization and analytical reproducibility 42.

Metal-metal core-shell nanoparticles can significantly enhance SERS detection performance by optimizing particle size and morphology for superior plasmonic enhancement. For instance, Mandavkar *et al.* 44 demonstrated two distinct AuPt core-shell nanostructures, both exhibiting substantially improved SERS activity compared to bare metallic counterparts. Beyond morphological variations, shell thickness also plays a critical role in governing SERS performance. Zhang's group 45 systematically investigated the correlation between shell thickness and SERS enhancement, revealing a synergistic mechanism. Their findings indicate that the thickness of ZIF-based shells exerts a significant influence on SERS signal intensity and reproducibility. In a parallel study, Su *et al.* 46 employed ZIF-8 as an encapsulating shell, observing that excessive shell thickness compromises synergistic enhancement capabilities, whereas an optimal ZIF-8 coating maintains elevated SERS activity while enhancing structural stability.

Moreover, gap-enhanced Raman tags (GERTs) represent emerging SERS probes with analytical, bioimaging, and theranostic utility. Their encapsulated reporter molecules resist environmental interference and particle aggregation while exhibiting enhanced signal fidelity through electromagnetic field amplification in metallic core-shell nanogaps 43, 47, 48 (Figure 4E). For instance, the Ye team engineered non-toxic GERTs as surgical tracers for precision sentinel lymph node (SLN) mapping 48. In rabbit models, GERTs enable intraoperative SLN identification within 10 min with a 4h differentiation window for secondary lymph nodes. Preoperative detection at 0.5 pM sensitivity provides minimally invasive positioning guidance. International Standard Organization (ISO)-compliant biosafety assessments confirm clinical translation potential for breast cancer SLN biopsy.

In conclusion, the plasmonic core of SERS tags consists of one or multiple plasmonic metal nanoparticles, which generate an intense electromagnetic field enhancement under incident light excitation via local surface plasmon resonance (LSPR). The types of plasmonic cores, apart from the three mentioned above, include many other categories. We can select the most suitable plasmonic core based on the specific application field.

### 3.2. Raman reporter molecules

RaRs constitute a critical component in the design of SERS tags. Within these systems, RaRs not only serve as signal transducers but also enhance performance by mitigating interference from intrinsic substrate signals. A key strategy for optimizing SERS nanotags involves the judicious selection of RaRs, which are categorized into two spectral regimes: the fingerprint region (<1800 cm^-1^) and the cellular silent region (1800-2800 cm^-1^). RaRs in the fingerprint region may exhibit spectral overlap with endogenous molecular vibrations, compromising analytical specificity. In contrast, RaRs occupying the cellular silent region generate distinct scattering peaks, as few endogenous molecules emit Raman signals in this spectral window, enabling unambiguous signal attribution and high signal-to-noise ratio (SNR) analyses.

The rational engineering of high-performance SERS nanotags requires meticulous consideration of the following parameters: (1) Affinity between RaRs and SERS substrates. Both EM enhancement (distance-dependent) and CM enhancement (reliant on covalent interactions) necessitate RaR immobilization on or near the plasmonic substrate surface. Strong RaR-substrate binding is essential to prevent desorption during functionalization or operational use. RaRs containing thiol groups or nitrogen-based ligands exhibit robust chemisorption to Au/Ag nanoparticles, making them ideal candidates. (2) Raman cross-section of RaRs. Larger molecular cross-sections correlate with stronger Raman signals, driving preferential selection of RaRs with high intrinsic polarizability. (3) Absorption wavelength of RaRs. Spectral alignment between RaR absorption and laser excitation wavelengths induces SERS, amplifying EFs by up to 100-fold and significantly boosting nanotag sensitivity. (4) Stability of RaRs. Fluctuations in RaR integrity can introduce spurious Raman signal variations, confounding the interpretation of target-dependent responses. Thus, chemically stable RaRs are imperative for reliable quantitative analysis. (5) Raman spectral profile of RaRs. For multiplexed detection or complex matrices, RaRs must exhibit sharp, non-overlapping Raman peaks to ensure spectral resolvability. Systematic optimization of RaRs based on these criteria significantly enhances the sensitivity, specificity, and reproducibility of SERS-based detection platforms.

#### 3.2.1 RaRs in the fingerprint region

Numerous conventional RaRs localized within the fingerprint spectral region, e.g., 4-mercaptobenzoic acid (4-MBA), 4-aminothiophenol (4-ATP), 4-nitrothiophenol (4-NTP), 4-mercaptophenol (4-MPH), rhodamine 6G (R6G), and malachite green isothiocyanate (MGITC), have been ubiquitously employed due to their commercial availability and facile functionalization 49. These molecules are typically immobilized on SERS-active substrates to engineer SERS nanotags or probes for diverse biosensing and bioanalytical applications. However, the spectral overlap between their Raman signatures and endogenous biomolecular vibrations (e.g., proteins, lipids) often compromises signal specificity, leading to ambiguities in data interpretation and reduced analytical accuracy.

RaRs play a crucial role in achieving chemical enhancement and multiplex detection. Recent advances focus on the strategies for enhancing Raman signal intensity, the use of responsive Raman reporters for signal readout, and the design of multiplexed Raman detection systems 50-53. For instance, the Stefan Harmsen group describes the development and preparation of a series of near-infrared-absorbing 2-thienyl-substituted chalcogenopyrylium derivatives specifically engineered for strong Au binding affinity. Upon adsorption onto Au NPs, these compounds form biocompatible SERS nanoprobes capable of attomolar detection limits, enabling ultra-sensitive multiplexed *in vivo* tumor and disease biomarker analysis 50. Ying Mao's group shows that activated microglia serve as reliable biomarkers for epileptogenic focus localization. Using a novel ratiometric Raman nanosensor (ultraHOCl), they visualized proinflammatory microglia in live epileptic mice with high precision, eliminating anesthesia-related artifacts. Additionally, ultraHOCl applied to human brain tissue excised from epilepsy patients achieved high sensitivity (94.89%) and specificity (93.3%) in distinguishing epileptic from non-epileptic regions 51. This approach offers an alternative intraoperative mapping strategy with the potential to improve surgical outcomes in epilepsy.

#### 3.2.2 RaRs in the cellular silent region

Advancements in Raman microscopy have enabled high-throughput acquisition of intense Raman signals and high-contrast imaging within cellular systems. Nevertheless, practical bioimaging analyses face challenges due to concurrent detection of Raman signals originating from both SERS nanoprobes and intrinsic cellular constituents. To address this limitation, Raman reporters featuring distinct vibrational modes within the cellular silent region (1800-2800 cm^-1^), such as alkynyl (C≡C), nitrile (C≡N), cyanide (CN⁻), thiocyanate (SCN/SCN⁻), carbonyl (C≡O), azide (N_3_⁻), and deuterium (C-D) groups, have emerged as superior candidates. These moieties exhibit minimal spectral interference from endogenous biomolecules, as cellular components lack significant Raman scattering in this spectral window. Their unique vibrational fingerprints and negligible photonic cross-talk render them indispensable for high-fidelity sensing and bioimaging under complex physiological conditions. Leveraging these advantages, researchers have developed SERS nanotags and probes incorporating silent-region reporters for multiplexed biosensing and *in situ* cellular imaging.

To maximize analytical performance, silent-region Raman reporters must satisfy the following requirements: (1) spectral distinctiveness. Reporters must generate robust, non-overlapping Raman peaks within the silent region to ensure high SNR, even at trace analyte concentrations. (2) chemical stability and biocompatibility. The reporter-substrate assembly must exhibit structural integrity and non-toxicity in biological environments throughout experimental workflows. (3) environmental responsiveness. Raman signals should selectively respond to target analytes without interference from non-specific interactions introduced by the nanoprobe architecture. (4) substrate compatibility. Reporter functionalization must not perturb the morphology, composition, plasmonic activity, or colloidal stability of the SERS substrate. Reporters incorporating C≡C, C≡N, CN⁻, SCN⁻, C≡O, N_3_⁻, or C-D functionalities largely fulfill these criteria, positioning them as optimal choices for enhancing SERS-based analytical platforms in biological matrices. The typical Raman signal molecules and their characteristic peaks are summarized in Table 4.

In conclusion, the identification capability of SERS nanotags is intrinsically linked to the selection of appropriate RaRs. When adsorbed onto the plasmonic metal core, these RaRs produce characteristic fingerprint spectra and exhibit signal amplification. By varying the chemical structure of RaRs molecules, a large library of SERS nanotags with distinct Raman-encoded signatures can be synthesized.

### 3.3. Protective coatings

When RaRs are directly exposed to complex biological media, molecules with a strong affinity for metal surfaces may compete with RaRs for binding sites, leading to the desorption of RaRs or their co-adsorption on nanoparticle surfaces. This phenomenon disrupts the spectral signatures of nanotags and significantly compromises the reliability of detection outcomes. Therefore, to mitigate spectral interference and signal fluctuations, nanoparticle surfaces functionalized with RaRs are typically coated with a protective layer 31. Additionally, the high ionic strength of biological media induces strong electrostatic interactions between unprotected nanoparticles, causing mutual attraction and aggregation. Aggregated nanoparticles exhibit markedly reduced stability, gradually precipitating over time. This sedimentation hinders their effective diffusion to target regions within biological systems, thereby impeding their intended functionality. Furthermore, the enlarged size of nanoparticle aggregates interferes with normal interactions between nanoparticles and cells, affecting cellular uptake, intracellular transport, distribution, and metabolism of nanoparticles, which may introduce detection biases or inaccuracies 67. The altered optical properties of aggregates also diminish SERS sensitivity by disrupting plasmonic hotspot formation, adversely affecting accurate SERS signal quantification. Consequently, protective coatings play a pivotal role in maintaining nanoparticle performance. Below, we elaborate on several widely utilized protective coatings.

#### 3.3.1. Inorganic coatings

Inorganic coatings, such as silica (SiO_2_) and metal oxides (e.g., TiO_2_), are commonly employed as protective materials for SERS nanotags. These coatings exhibit exceptional chemical stability, thermal resistance, and mechanical robustness, effectively preventing oxidation and aggregation of metal nanoparticles while shielding RaRs from degradation. Beyond enhancing nanotag stability, inorganic coatings ensure reliable performance in complex biological environments, making them indispensable for applications requiring long-term durability and consistent signal output.

##### 3.3.1.1. Silica

Silica (SiO_2_), as a protective coating for SERS nanotags, exhibits exceptional chemical stability, tunable thickness, and favorable biocompatibility. It effectively prevents oxidation and aggregation of metal nanoparticles while maintaining optical transparency and signal enhancement capabilities, making it one of the most widely adopted coating materials.

The facile surface functionalization of silica further broadens its applicability across diverse scenarios. For instance, Yu *et al.* designed a silica-encapsulated Au core-satellite (CS@SiO_2_) nanotag capable of generating stable SERS signals 68. This nanotag achieved highly sensitive detection of SARS-CoV-2. Bock *et al.* proposed that Au-assembled nanostructures with controllable nanogaps could exhibit intense SERS signals from multiple hotspots, representing a breakthrough in the field 69. They synthesized SiO_2_@Au@Au NPs via a seed-mediated growth method, creating near-infrared (NIR) SERS nanoprobes. By modulating the concentration of Au precursors, the size of Au NPs and the interparticle gaps on the silica surface were precisely controlled to optimize SERS hotspot formation. Scarpitti *et al.* 70 developed silica-encapsulated GERTs for single-particle Raman imaging and quantitative analysis in live cells. Validated by single-particle inductively coupled plasma mass spectrometry (spICP-MS), this approach not only quantified cellular uptake but also imaged subcellular distribution and assessed nanoparticle stability.

##### 3.3.1.2. Titanium dioxide

Titanium dioxide (TiO_2_) exhibits superior optical transparency and chemical stability, effectively protecting metallic nanoparticles from oxidation and aggregation while enhancing the stability of SERS signals. In coating applications, the inherent photocatalytic properties of TiO_2_ enable pollutant degradation or enhanced reactivity under illumination, thereby broadening the application scope of SERS nanotags. For instance, Chen *et al.* 71 engineered a novel spherical nanocage reactor (de-Au@mTiO_2_) featuring mesoporous TiO_2_ shells encapsulating Au nanoparticles of varying sizes, creating abundant nanogaps and shared hotspots to significantly amplify SERS performance. This material was successfully applied in the photocatalytic cleavage of NADH (a critical enzyme in tumor metabolism), with SERS revealing molecular-level mechanisms, thereby providing novel strategies for suppressing tumor cell activity.

Furthermore, as a widely used semiconductor material, TiO_2_-based SERS substrates leveraging semiconductor metal oxide nanomaterials have gained extensive adoption across multiple disciplines due to their cost-effectiveness, exceptional stability, and favorable biocompatibility. For example, Chen *et al.* 72 proposed an innovative strategy for fabricating TiO_2_/cellulose nanofibril (CNF) films as SERS substrates via directional assembly. A 10 nm-thick TiO_2_/CNF film deposited on indium tin oxide (ITO) exhibited a remarkable enhancement factor of 1.79×10^6^, enabling ultrasensitive detection of 4-mercaptobenzoic acid as low as 10 nM. This performance enhancement was attributed to the synergistic modulation of the CNF network's templated morphology and the crystalline state of TiO_2_.

#### 3.3.2. Organic coatings

Organic coatings represent a widely utilized class of protective materials in SERS nanotags, primarily including proteins 73, polymers 74, 75, and liposomes 76. These coatings exhibit excellent biocompatibility and functionality, effectively preventing nanoparticle aggregation and enhancing their stability in biological environments 67, 77. Organic coatings not only preserve the stability of SERS signals but also provide critical support for their applications in complex biological systems. Next, we will mainly introduce several commonly used ones.

##### 3.3.2.1. Proteins

**Figure 5:**
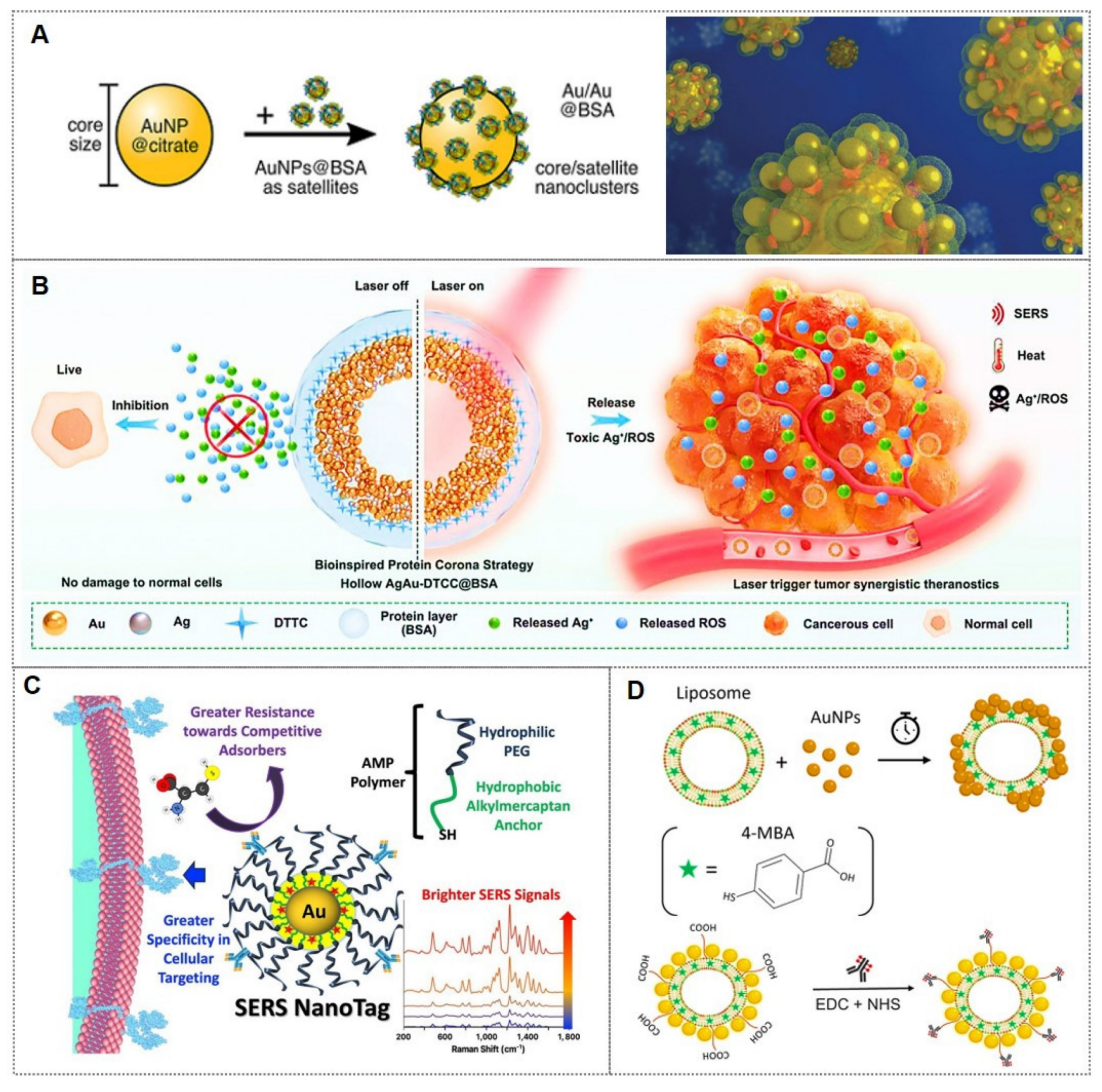
(A) Schematic illustration of small protein-coated NPs (satellites) selectively adsorbing onto protein-free cores to form nanoclusters. Reproduced with permission from Ref. 73. Copyright 2024, American Chemical Society. (B) Schematic illustration of Ag-hybridized hollow Au nanoshells with bioinspired protein coronas, enabling SERS imaging and tumor-specific phototherapy activation. Reproduced with permission from Ref. 78. Copyright 2021, Elsevier. (C) Schematic illustration of AMP-coated SERS nanotags utilizing hydrophobic locking for enhanced brightness, stability, and cellular targeting. Reproduced with permission from Ref. 75. Copyright 2024, Elsevier. (D) Schematic illustration of LipoGold SERS tag synthesis via EDC/NHS-mediated antibody conjugation on liposome-AuNP hybrids. Reproduced with permission from Ref. 76. Copyright 2024, Nature Communications.

Proteins rapidly adsorb onto the surfaces of metallic nanoparticles, forming a “protein corona”. This corona comprises multiple protein species, with its composition and thickness determined by protein concentration, nanoparticle surface properties, and environmental conditions. Consequently, protein coatings on metallic nanoparticles effectively mitigate particle aggregation, thereby optimizing nanoparticle performance. Among the most commonly employed protein coatings is bovine serum albumin (BSA), which is favored for its low cost, accessibility, and biocompatibility. For instance, the Holler group 73 utilized BSA to assemble superstructures with varying core sizes, employing an optical isotropic model system featuring a spherical fiber core/satellite architecture (Figure 5A). Using mercaptobenzoic acid (MBA) as the analyte, they achieved a SERS detection limit of 10^-7^ M. Their work demonstrated the importance of colloidal stability in measurements, elucidated theoretical principles of hotspot formation, and provided guidelines for designing SERS sensing probes.

Similarly, the Zhou research 78 developed a bioinspired protein corona platform by assembling hollow Ag-Au nanoshells conjugated with DTTC Raman tags and BSA (designated as AgAu-DTTC-BSA). This design effectively minimized ROS production triggered by silver ions, safeguarding healthy cells and tissues, while allowing laser-induced activation at targeted tumor regions (Figure 5B). These nanoshells demonstrated remarkable LSPR effects, which endowed them with highly efficient and stable photothermal conversion capabilities under laser irradiation, coupled with enhanced SERS activity. Furthermore, the biocompatible hollow AgAu-DTTC-BSA nanocomposites exhibited superior therapeutic efficacy against colorectal cancer through precise SERS imaging-guided photothermal therapy for solid tumor ablation, synergistically complemented by the controlled release of cytotoxic Ag⁺ ions and ROS generation.

##### 3.3.2.2. Polymers

Polymers represent the most prevalent type of organic coatings, attributed to their exceptional biocompatibility and colloidal stability, which effectively prevent nanoparticle aggregation and enhance their dispersibility in complex biological environments. Furthermore, polymer coatings can be functionalized through surface modifications, thereby expanding the application potential of SERS nanotags in biomedical detection and therapeutics.

Polyethylene glycol (PEG) is widely employed due to its high hydrophilicity, chemical inertness, and antifouling properties, which facilitate prolonged circulation in biological systems. For instance, the Dirisala group 74 developed hepatic sinusoid-selective coatings based on linear or two-arm PEG-conjugated oligolysine (OligoLys). This transient coating effectively circumvented hepatic sinusoidal clearance of non-viral and viral gene vectors, significantly enhancing their gene transfection efficiency in target tissues. Similarly, Lane *et al.* 75 reported a novel class of bright and stable SERS nanotags utilizing alkylthiol-PEG (AMP) polymers. The amphiphilic structure and thiol anchoring groups of AMP enabled strong adsorption onto gold nanoparticles (Figure 5C). The internal hydrophobic layer encapsulated Raman reporter molecules, while the external hydrophilic layer prevented competitive adsorption of other molecules. High AMP grafting density improved cellular target selectivity. This configuration provided a more favorable dielectric environment, yielding brighter nanotags and enhanced sensitivity in cellular detection.

##### 3.3.2.3. Lipids

Lipids are widely recognized as an exceptional organic coating due to their inherent biocompatibility and capacity for self-assembly on nanoparticle surfaces. In the context of imaging, Cardellini *et al.* 76 developed the LipoGold tag platform, wherein gold nanoparticles self-assemble into clusters on lipid vesicles, significantly amplifying Raman reporter signals (Figure 5D). After optimizing nanoparticle concentrations, optimal SERS enhancement was achieved, and structural characterization of the platform was performed. The LipoGold tags were successfully functionalized with antibodies to detect intracellular GM1 variations, enabling discrimination between healthy donors and patients with GM1 gangliosidosis. This advancement underscores the platform's potential for sophisticated applications in SERS-based probes.

#### 3.3.3. Composite coatings

In addition to the aforementioned coating types, composite coatings (specifically inorganic-organic hybrid coatings) have garnered significant attention. Among these, metal-organic framework (MOF) coatings are particularly prominent. Owing to their high porosity, structural diversity, biocompatibility, and superior stability, MOFs have been extensively applied in diverse fields, including the encapsulation of SERS nanotags. These attributes enable MOFs to enhance the affinity of SERS nanotags toward analytes, provide stronger SERS enhancement, and protect the nanotags from corrosion, positioning them as a high-performance coating.

**Figure 6:**
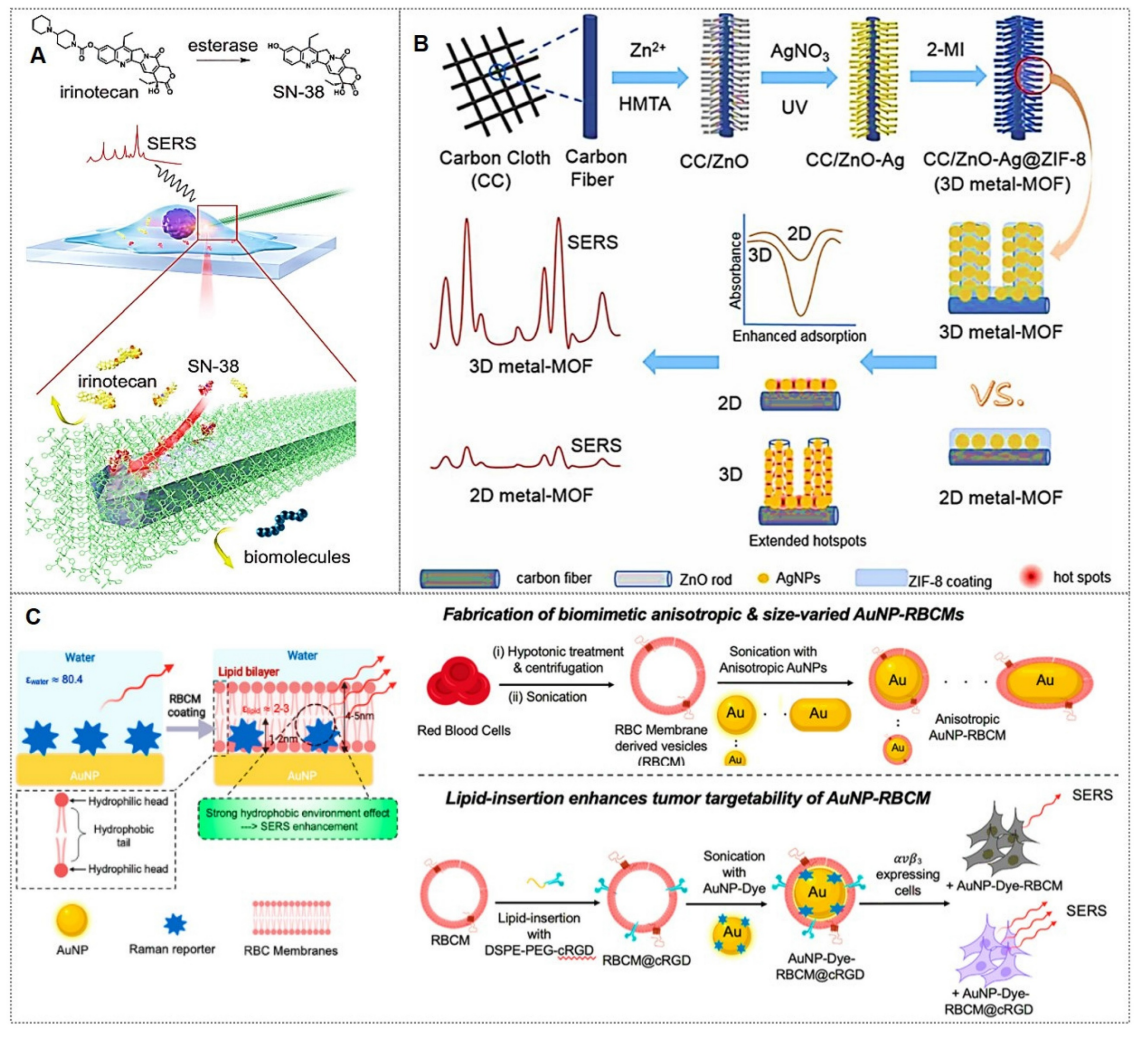
(A) MOF-coated nanowire endoscopy for intracellular selective detection. Reproduced with permission from Ref. 79. Copyright 2023, Wiley. (B) 3D noble metal-MOF composite enabling pesticide capture and detection. Reproduced with permission from Ref. 80. Copyright 2021, Elsevier. (C) Biofunctionalized anisotropic Au NPs coated with RBCM vesicles. Reproduced with permission from Ref. 83. Copyright 2022, American Chemical Society.

Zeolitic imidazolate framework-8 (ZIF-8) is the most widely utilized MOF for coating shells due to its facile synthesis, exceptional stability, and low cytotoxicity. For example, Zhang *et al.* 79 developed an endoscopic probe coated with ZIF-8-encapsulated silver nanowires for real-time monitoring of the metabolism of the anticancer drug irinotecan (Figure 6A). The probe successfully tracked the conversion of irinotecan to SN-38 and monitored its intracellular distribution in real-time, demonstrating the potential of MOF-coated probes for specific drug metabolism studies. Similarly, Pu *et al.* 80 developed a 3D SERS platform (CC/ZnO-Ag@ZIF-8) by growing ZnO nanorods on carbon cloth, functionalizing them with Ag NPs, and encapsulating the structure in a ZIF-8 layer. This substrate achieved ultrasensitive pesticide monitoring (Figure 6B). The ZIF-8 coating improved stability and anti-interference capability, and the substrate was successfully applied for environmental analysis.

Beyond the widely studied coatings mentioned above, emerging coating types with substantial development potential include graphene 81, 82, erythrocyte membranes 83, 84, and others. For instance, the Sun group 81 synthesized GO nanocomposites decorated with Fe_3_O_4_@Au@Ag NPs exhibiting high SERS activity and stability, which enables real-time monitoring of Doxorubicin (DOX) release dynamics through temporal variations in SERS spectra. The Gao team 82 developed a background-free SERS chip with a sandwich architecture for reliable multiplex bacterial detection and photothermal eradication. Notably, the Nie group 83 introduced a simple but effective biomimetic strategy by cloaking colloidal SERS nanoparticles with erythrocyte membranes (RBCM) (Figure 6C). Building on this, subsequent research 84 developed dual-modal gold nanostars (Au NS) for combined SERS and photoacoustic (PA) tumor detection in *ex vivo* tissues. These RBCM-coated agents improved tissue penetration and allowed Raman-PA correlation analysis to locate hidden tumors. Each coating type imparts distinct functionalities to SERS nanotags, enabling their adaptation to diverse application scenarios.

In conclusion, the protective shell in SERS nanotags fulfills four critical roles: (1) preventing RaRs desorption from the nanoparticle surface; (2) mitigating potential signal contamination from ambient impurities; (3) reducing the biotoxicity of metallic nanoparticles; (4) suppressing plasmonic coupling between adjacent nanoparticles. The choice of protective shell material is application-dependent, with common options including biocompatible polymers, inorganic coatings, or biomolecules.

### 3.4. Probing molecules

To enhance the sensitivity, targeting capability, and multifunctionality of nanotags, the final step in nanotag fabrication typically involves surface conjugation of probing molecules. These molecules exhibit diverse functionalities, and herein we focus on the most widely utilized types.

#### 3.4.1. Antibodies

**Figure 7:**
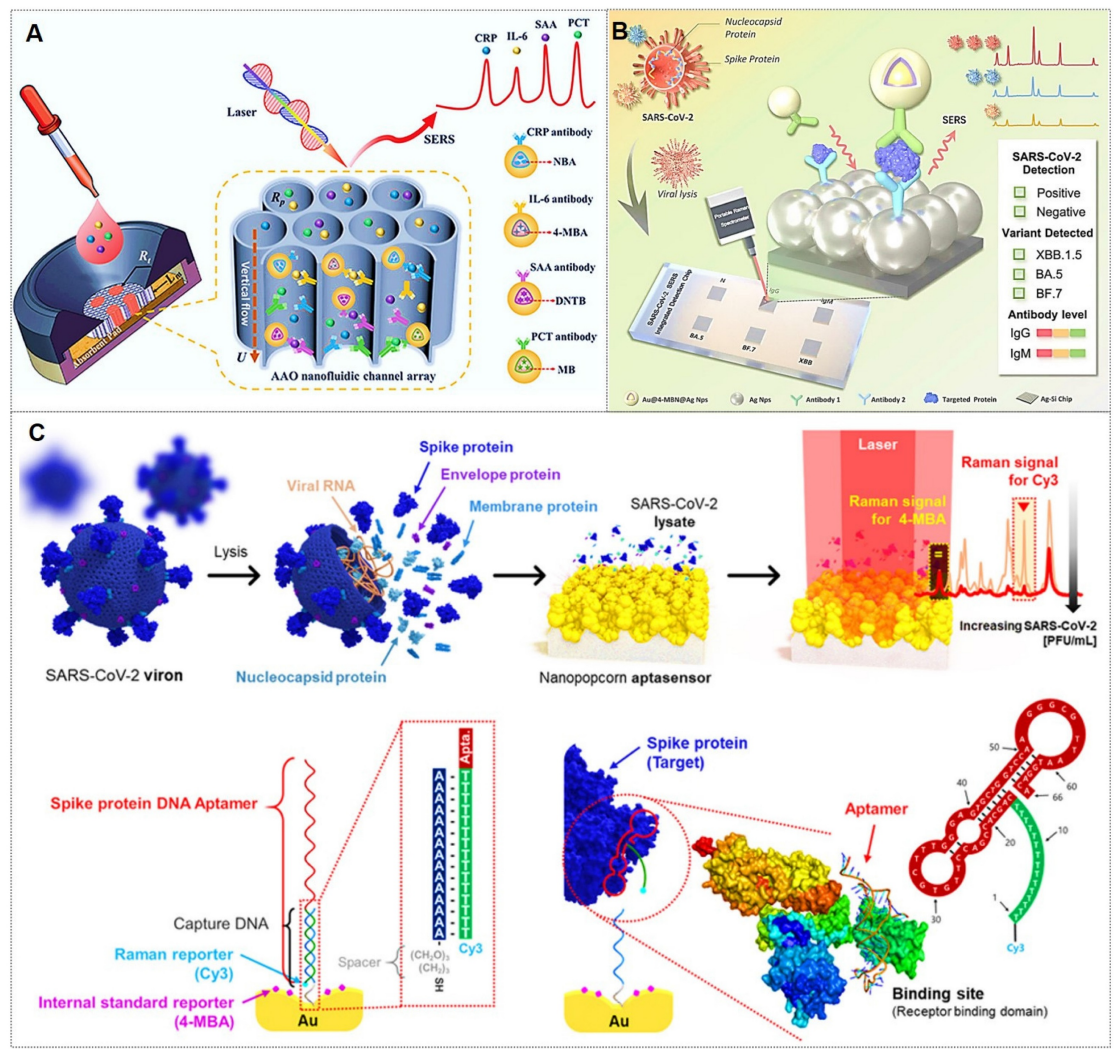
(A) Nanoporous AAO-based VFA platform for multiplex detection of four inflammatory biomarkers using Raman dye-encoded core-shell SERS nanotags. Reproduced with permission from Ref. 85. Copyright 2020, Wiley. (B) Multifunctional SERS chip enabling antigen/antibody detection of SARS-CoV-2. Reproduced with permission from Ref. 86. Copyright 2025, Elsevier. (C) SERS aptasensor for quantitative analysis of SARS-CoV-2 viral load. Reproduced with permission from Ref. 87. Copyright 2021, American Chemical Society.

Antibodies represent the most prevalent class of targeting molecules due to their high specificity and affinity for recognizing target analytes, thereby enabling precise targeting 85, 86. For instance, Chen *et al.* 85 pioneered the integration of vertically aligned anodic aluminum oxide (AAO) membranes with ultrasensitive SERS nanotags in a vertical flow assay (VFA) for multiplex detection of four inflammatory biomarkers (Figure 7A). The high surface area of AAO and the exceptional sensitivity of SERS nanotags enabled biomarker quantification across a broad linear dynamic range, demonstrating the potential for point-of-care diagnosis and management of inflammatory diseases. The Lin research group 86 designed a multi-channel SERS nanochip for simultaneous detection of SARS-CoV-2 Omicron proteins (N/S) and antibodies (IgG/IgM). This platform achieved subtype-specific identification of Omicron variants (BA.5, BF.7, XBB.1.5) with a detection limit of 0.16 pg/mL (Figure 7B), offering a rapid POCT solution for viral diagnosis, variant tracking, and post-infection immunity assessment. Despite their superior specificity, antibodies exhibit environmental sensitivity, with performance being influenced by pH, temperature, and ionic strength. Consequently, meticulous control of fabrication conditions is critical during nanotag assembly.

#### 3.4.2. Aptamers

Aptamers are synthetic nucleic acids that bind target molecules with high specificity, typically selected through systematic evolution of ligands by the exponential enrichment (SELEX) technique 87. These functional strands have enabled advanced biomedical detection tools. For instance, the Jaebum Choo group 87 created a SERS aptasensor using spike protein DNA aptamers as receptors and self-assembled gold nano-popcorn as substrates. This platform quantitatively detects SARS-CoV-2 by measuring SERS signal shifts from aptamer-virus binding, achieving results within 15 minutes and a detection limit below 10 PFU/mL (Figure 7C).

Beyond cancer biomarker detection, aptamer-based nanotags are also applicable to viral detection 87, 88 and drug monitoring 89. Despite these merits, aptamers may suffer from interference in complex biological matrices, necessitating optimized conjugation strategies to enhance stability and signal intensity during fabrication.

#### 3.4.3. Environmentally responsive molecules

Environmentally responsive molecules are chemical entities that undergo structural, physical, or electronic state changes in response to external stimuli such as pH, temperature, light, or redox conditions. These molecules exhibit reversible or irreversible alterations in physicochemical properties under specific environmental triggers, thereby modulating their optical, electrical, or mechanical behaviors.

The SERS nanotags represent a cutting-edge class of nanosensors that integrate plasmonic nanoparticles with molecular reporters. A key feature of these nanotags is the incorporation of environmentally responsive molecules, which undergo conformational or spectroscopic changes in response to specific external or internal stimuli. These molecules are predominantly engineered to detect variations in pH, temperature, light exposure, or redox potential. The functionality of these molecules relies on their stimulus-dependent properties, such as shifts in vibrational modes, charge transfer efficiency, or plasmonic coupling. By capitalizing on these dynamics, SERS nanotags achieve ultra-sensitive, multiplexed detection of analytes in complex matrices, including biological fluids, ecological samples, and food. This capability is pivotal for real-time monitoring of disease biomarkers (e.g., cancer-related enzymes in tumor microenvironments).

Notably, Table 5 summarizes representative environmentally responsive molecules and their application scenarios. Examples include: (1) pH-responsive molecules (e.g., 4-MBA: 4-mercaptobenzoic acid; 4-MPY: 4-mercaptopyridine; BCCDP: benzyl 4-(9-(6-cyanopyridin)-9H-carbazole) 5-(1,2-dithiother) pentanoate; 4-MPBA: 4-Mercaptophenylboronic acid); (2) Temperature-responsive molecules (e.g., PNIPAAm: poly [N-isopropylacrylamide-co-N, N'-methylene bis(acrylamide); Resazurin); (3) Redox-active compounds (e.g., 2-MBQ: 2-Mercaptobenzoquione; HQ: ortho-mercaptohydroquinone); (4) Light-active compounds (e.g., DTTC: 3,3'-diethylthiatricarbocyanine iodide). These innovations underscore the critical contributions of environmentally responsive molecules to expanding SERS applications in biomedicine. Future developments may focus on integrating multi-stimuli-responsive systems and machine learning algorithms to improve specificity and field deployability.

In conclusion, selective targeting of specific tissues or regions is achieved by functionalizing the nanotags with biorecognition ligands, such as antibodies, aptamers, peptides, or proteins, which enable precise molecular recognition and binding. This is the foundation for the selective analysis, detection, and application of SERS tags.

## 4. Applications of SERS nanotags in biomedicine

**Figure 8:**
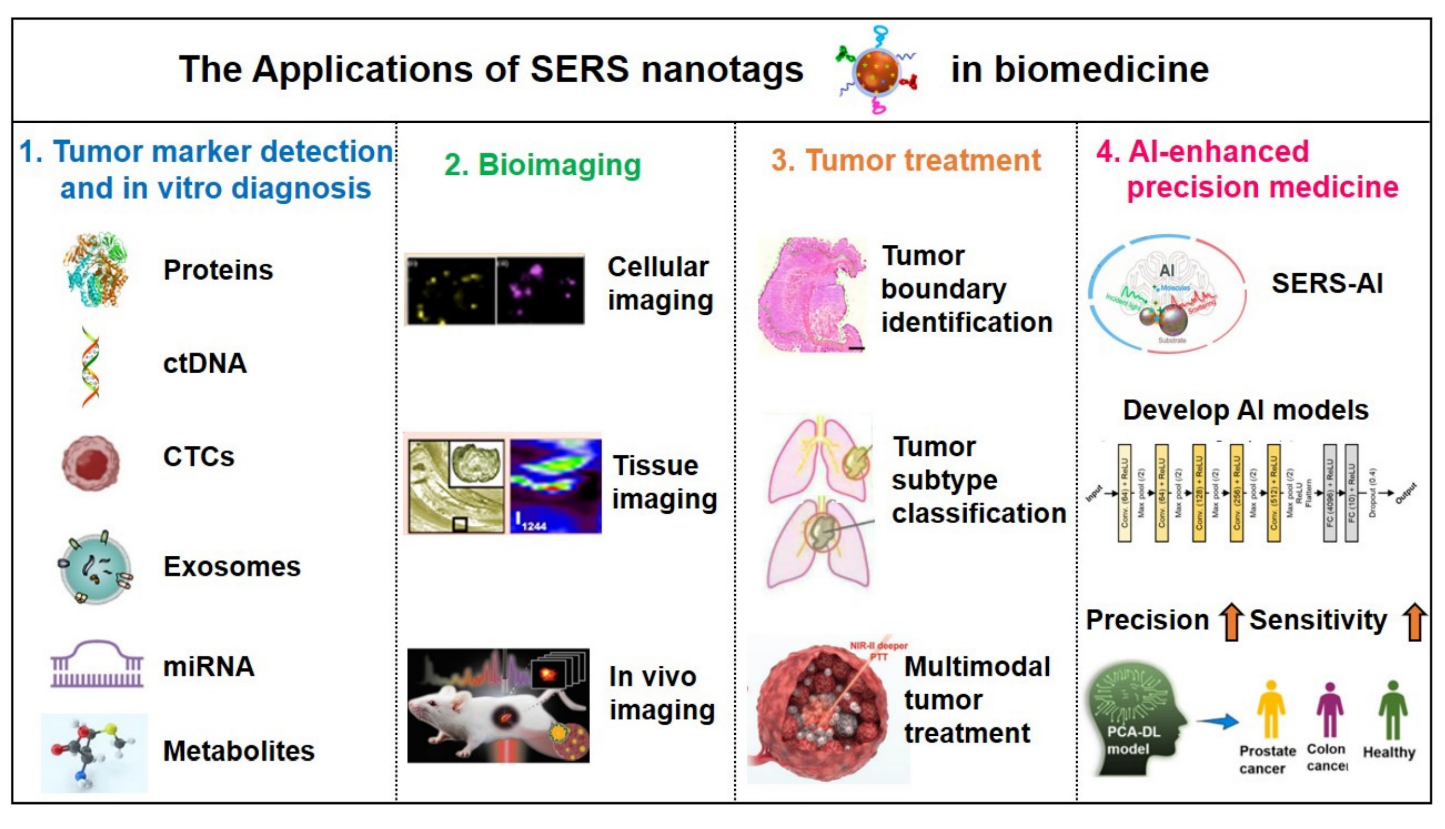
The framework of the four main applications of SERS nanotags in biomedicine.

As previously mentioned, SERS nanotags exhibit prominent advantages, including high sensitivity, specificity, stability, and environmental responsiveness, thereby demonstrating extensive applications across various biomedical domains. The main application areas include: (1) tumor marker detection and *in vitro* diagnosis; (2) bioimaging; (3) tumor treatment; (4) Artificial intelligence (AI)-enhanced tumor diagnosis and treatment. The general framework diagram is shown in Figure 8. Next, we will introduce each of these four aspects.

### 4.1. The tumor marker detection and *in vitro* diagnosis

Malignant tumors represent one of the most prominent and severe global health challenges. Conventional tumor screening methodologies, such as imaging examinations and tissue biopsies, face limitations in diagnostic accuracy and inherent procedural risks. With advancements in research technologies, it has been established that distinct tumor types typically express specific biomarkers, providing novel perspectives for tumor screening. Tumor biomarkers refer to characteristic biological molecules, either secreted by malignant cells or generated through host responses, that correlate with tumorigenesis and progression. These biomarkers, detectable in tumor tissues or body fluids, hold critical scientific significance for early tumor screening and therapeutic monitoring 98.

#### 4.1.1. Conventional tumor markers in body fluids

In recent years, the detection of biomarkers in body fluids has replaced the traditional detection of tissue biomarkers and has become a critical diagnostic modality for malignancies. This approach primarily evaluates disease progression through the identification and concentration analysis of specific biomarkers in biofluids, and conventional fluid-phase detection targets proteinaceous compounds. Beyond establishing SERS-integrated machine-learning diagnostic platforms for tumor biomarkers, researchers have optimized sample processing, substrate design, and nanoprobe synthesis to enhance the sensitivity and accuracy of SERS detection.

**Figure 9:**
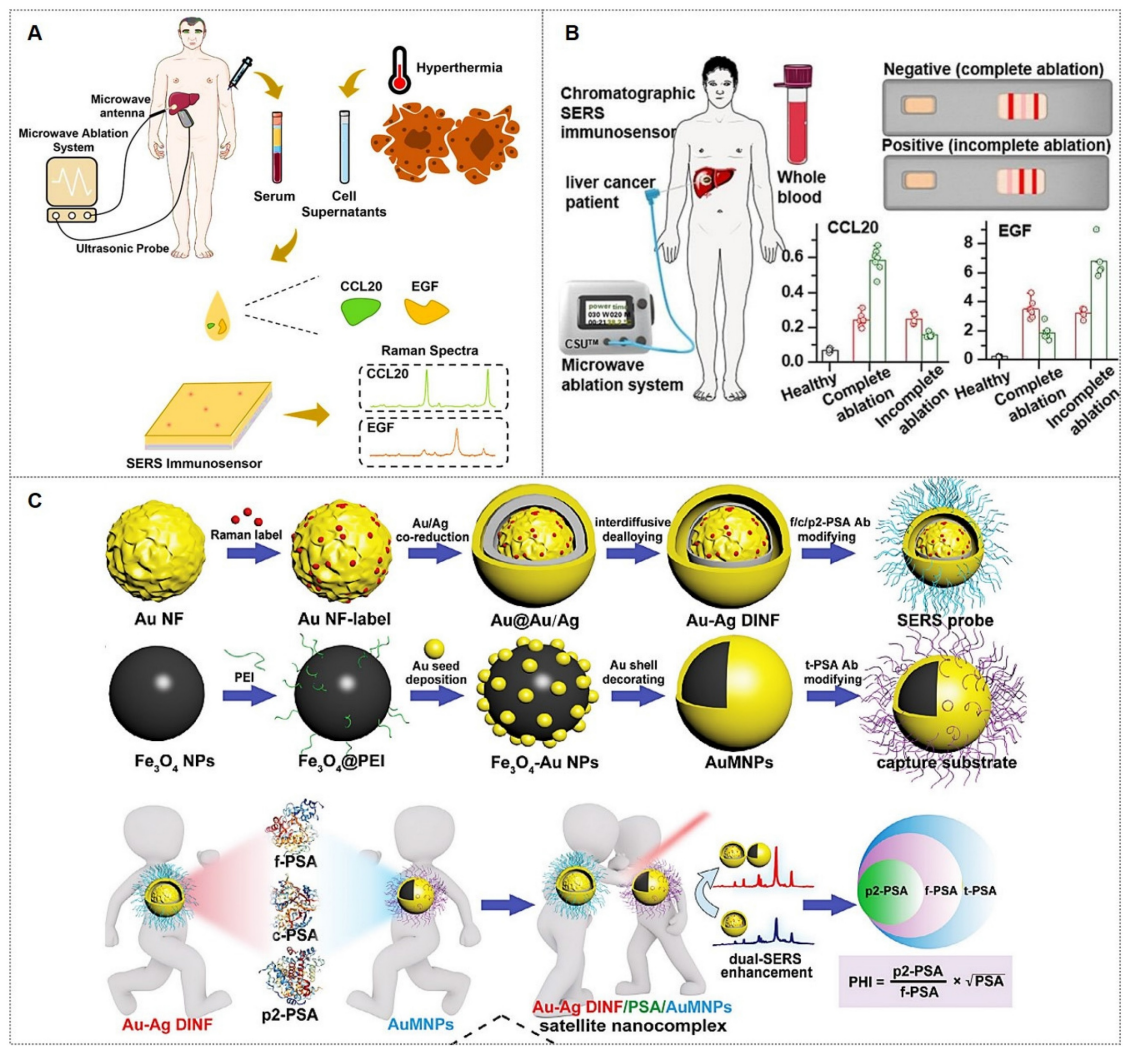
(A) Quantitative SERS detection of serum protein biomarkers for evaluating tumor microwave ablation efficacy. Reproduced with permission from Ref. 99. Copyright 2024, Elsevier. (B) SERS lateral flow strip for noninvasive monitoring of microwave ablation outcomes in unresectable hepatocellular carcinoma. Reproduced with permission from Ref. 100. Copyright 2024, Elsevier. (C) Synthesis of Au-Ag DINF and Au MNP substrates with SERS satellite strategy for multicomponent PSA detection in PHI-based PCa diagnosis. Reproduced with permission from Ref. 101. Copyright 2022, Wiley.

For protein biomarker detection via SERS, sandwich immunoassays employing aptamers or antibodies dominate as the primary strategy to achieve target-specific binding. Microwave ablation (MWA) serves as a critical therapeutic intervention for hepatocellular carcinoma (HCC), yet it lacks biomarkers for ablation efficacy assessment. As depicted in Figure 9A, Ouyang *et al.* 99 identified CCL20 and EGF as novel biomarkers for ablation evaluation through cellular assays and clinical serum analysis, subsequently developing a high-sensitivity sandwich SERS immunosensor. This sensor achieved detection limits of 0.082 pg/mL (CCL20) and 0.096 pg/mL (EGF) within linear ranges of 0.1 pg/mL to 1 ng/mL, showing strong concordance with ELISA results. Similarly, Su *et al.* 100 reported a SERS-lateral flow strip (LFS) immunosensor for simultaneous quantification of CCL20 and EGF proteins, enabling rapid noninvasive evaluation of MWA outcomes in HCC patients (Figure 9B).

Beyond antibodies, aptamers, single-stranded DNA (ssDNA), or RNA exhibit comparable target-specific affinity while offering unique biosensing advantages: broader target diversity, smaller molecular footprint, minimal immunogenicity/toxicity, cost-effectiveness, and facile chemical modification. These features have driven their widespread adoption in SERS-based immunoassays. For example, Zhou *et al.* 101 developed a dual SERS-satellite immunoassay to simultaneously detect multiple PSA variants (fPSA, cPSA, p2PSA). This platform introduced a novel PSA-mediated Prostate Health Index (PHI) calculation, outperforming traditional tPSA/fPSA% ratios in PCa specificity (Figure 9C). By combining immuno-nanoassemblies with dual SERS amplification, the system enables comprehensive PCa screening and prognosis monitoring.

#### 4.1.2. Novel tumor markers in liquid biopsy‌

**Figure 10:**
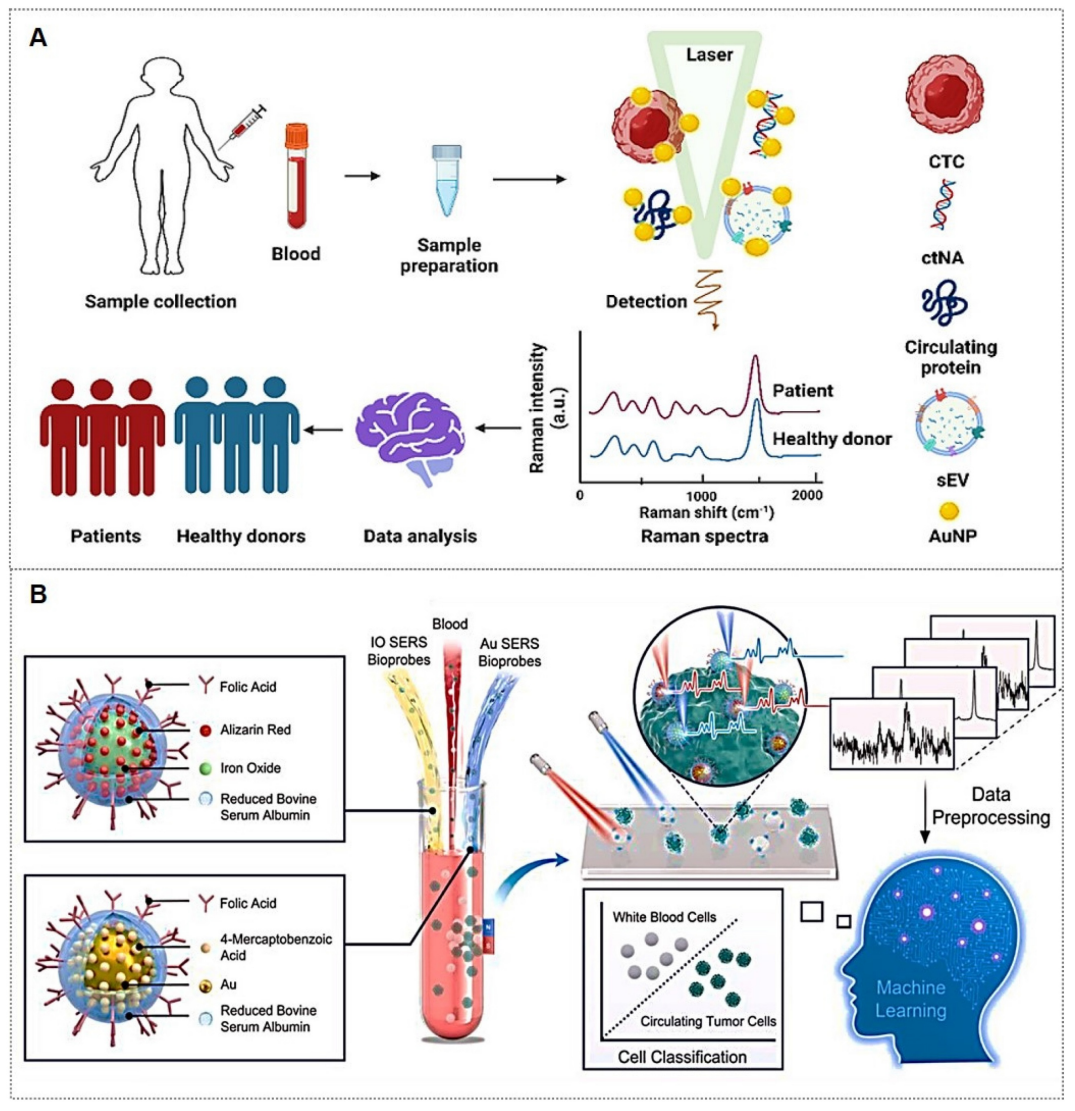
(A) ‌Liquid Biopsy SERS Assay‌: Analyte-bound SERS substrates generate intrinsic Raman spectra for cancer detection via machine learning. Reproduced with permission from Ref. 103. Copyright 2024, Springer. (B) Dual-Modal SERS Detection‌: Integrates machine learning for CTC analysis. Reproduced with permission from Ref. 108. Copyright 2025, Elsevier.

Traditional detection of tumor markers in body fluids has been largely confined to single-cancer analysis. The discovery of novel tumor biomarkers has expanded the scope of liquid biopsy and propelled advancements in liquid biopsy technologies. Current novel tumor biomarkers include circulating tumor cells (CTCs), cell-free DNA (cfDNA), circulating tumor DNA (ctDNA), exosomes, and microRNAs (miRNAs). Liquid biopsy enables analysis of tumor genomic data from bodily fluids of cancer patients, providing critical insights into tumor progression, staging, heterogeneity, and genetic mutations 102. The multiplexing capability of SERS positions it as an ideal biosensing platform for such applications (Figure 10A) 103, 104. The integration of liquid biopsy with SERS has significantly enhanced detection sensitivity and specificity, establishing SERS as a pivotal technology for biomedical analysis of diverse tumor biomarkers.

##### 4.1.2.1. ctDNA‌

ctDNAs are DNA fragments released into the peripheral bloodstream following tumor cell lysis. Detection of mutation loci in ctDNA from bodily fluids holds significant implications for guiding precision therapeutic strategies 105-107.

ctDNA has emerged as a highly sensitive biomarker for early-stage gastric cancer (GC) detection and prognostic evaluation. For GC, the Cao research team 105 developed a pump-free microfluidic chip that integrates catalytic hairpin assembly (CHA) and hybridization chain reaction (HCR) for SERS-based detection of PIK3CA E542K and TP53 ctDNAs. With six parallel units, it achieves attomolar sensitivity (1.26 aM for PIK3CA, 2.04 aM for TP53) in 13 minutes. For NSCLC, they engineered a dual-signal amplification strategy combining CHA and pump-free SERS microfluidics that detects BRAF V600E and KRAS G12V ctDNAs in 5 minutes, demonstrating exceptional selectivity, reproducibility, and homogeneity 106. This platform offers rapid, high-throughput ctDNA quantification with significant clinical potential for cancer diagnosis.

##### 4.1.2.2. CTCs‌

CTCs, defined as tumor cells shed from primary lesions into peripheral blood with high metastatic potential and viability, serve as critical tools for early cancer diagnosis, prognosis evaluation, and postoperative monitoring.

For instance, Zhang *et al.*‌ 108 developed a detection strategy integrating encoded SERS probes and machine learning models for the identification of CTCs (Figure 10B). This simple yet efficient approach provides a novel methodology for CTC detection, demonstrating significant implications for cancer diagnostics. Xu *et al.* 109 integrated a microfilter separation method with SERS probes to achieve simultaneous isolation and detection of CTCs in peripheral blood. Leveraging differences in size and deformability between CTCs and blood cells, CTCs were rapidly isolated and captured using microfilters within minutes. This approach reduced the total CTC detection time to under 1.5 hours, achieving a LOD of 2 cells/mL. Similarly, He *et al.* 110 synthesized surface-defective octahedral Ag_2_O nanoparticles exhibiting excellent biocompatibility, remarkable SERS enhancement, and enabling the detection of CTCs in peripheral blood samples from two HCC patients with a detection limit of 1 cell/mL. Li *et al.*‌ 111 proposed a novel strategy for non-destructive isolation/enrichment and ultrasensitive SERS-based CTC counting via aptamer recognition and rolling circle amplification (RCA). This approach holds promising potential for detecting CTCs in blood, offering a robust tool for liquid biopsy-based analysis of extremely rare CTCs in complex peripheral blood samples.

##### 4.1.2.3. Exosomes‌

**Figure 11:**
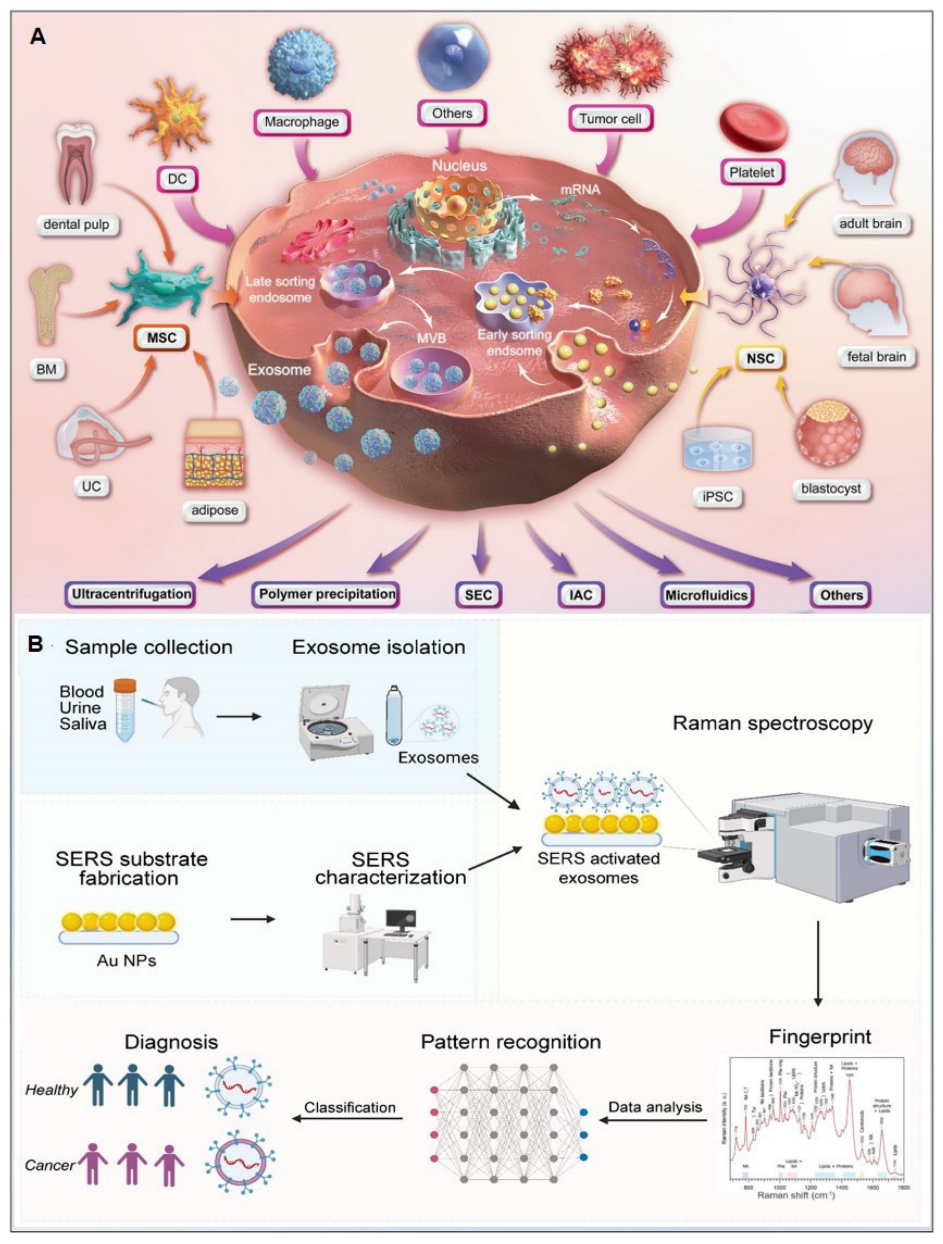
(A) ‌The production and purification of Exosomes‌. Abbreviations: BM (bone marrow), DC (dendritic cell), MSC (mesenchymal stem cell), UC (umbilical cord), NSC (neural stem cell), iPSC (induced pluripotent stem cell), MVB (multivesicular body), SEC (size-exclusion chromatography), IAC (immunoaffinity chromatography). Reproduced with permission from Ref. 112. Copyright 2024, Springer Nature. (B) Illustration of exosome analysis for cancer diagnosis using SERS. Reproduced with permission from Ref. 114. Copyright 2024, Ivyspring.

Exosomes carry molecular information (e.g., proteins, nucleic acids, and metabolites) from parental cells and are encapsulated by a lipid bilayer membrane, which protects their biomarkers from enzymatic degradation in the extracellular environment. Functioning as efficient mediators of intercellular communication, exosomes play critical roles in tumor metastasis and immune regulation. Compared to other circulating biomarkers, such as CTCs, nucleic acids, and metabolites, exosomes exhibit high abundance and superior stability due to their significantly larger size (Figure 11A) 112, 113. Furthermore, the phosphorylation status of exosomal proteins is closely associated with tumor progression, positioning exosomes as promising biomarkers for cancer diagnostics. The workflow of Exosome analysis for cancer diagnosis using SERS includes: sample collection and exosome isolation; fabrication and characterization of SERS substrate; spectroscopic data collection via SERS; exosome classification and cancer diagnosis based on SERS patterns (Figure 11B) 114.

Exosomal liquid biopsies provide a noninvasive approach for the early identification of malignancies and dynamic disease tracking. Ongoing clinical investigations aim to confirm the clinical utility of exosomal biomarkers in improving diagnostic precision and forecasting therapeutic outcomes 115. For instance, Song group developed a multivalent aptamer-linked tetrahedral DNA (MATD)-assisted catalytic hairpin assembly (CHA) SERS assay, enabling ultrasensitive (detection limit: 2.98×10^3^ particles/mL, ~6 exosomes/2 μL) and specific detection of cancer-derived exosomes in 40 minutes 116. The Fang group introduced a label-free SERS method to analyze extracellular vesicles (EVs) biophysical heterogeneity by optimizing nano-enhanced particle (NEP) sizes. PCA-based classification of normal vs. cancerous exosomes improved accuracy from 91.2% to 95.1%. This strategy enhances understanding of EV surface properties, size, and morphology, with broad applications in functional research 117. The Cao group introduces ‌Gemini‌, a dual-signal SERS platform enabling ultrasensitive, cross-category quantification of pyruvate (metabolite) and lactate dehydrogenase B (LDHB, protein) in plasma-derived exosomes. This design yields two distinct Raman signals for ‌ratiometric, interference-free detection‌, achieving limits of 2.415 μM (pyruvate) and 0.032 ng/mL (LDHB). When paired with a ‌support vector machine (SVM) classifier‌, Gemini distinguishes acute coronary syndrome-induced sudden cardiac death (ACS-SCD) patients from healthy controls with ‌85% accuracy‌ (90% sensitivity, 80% specificity; AUC = 0.82 vs. 0.79 for conventional methods) 118.

##### 4.1.2.4. miRNAs

In recent years, aberrant miRNA expression has been implicated in various pathologies, including cancers, genetic disorders, and neurological diseases, establishing miRNAs as novel biomarkers 119. Due to their low endogenous abundance, the development of miRNA detection technologies commonly relies on signal amplification strategies and integration of SERS with other sensing modalities. For instance, Shen *et al.* 120 achieved simultaneous detection of miR-155 and miR-21 using a dual-target recognition probe (DRP) based on a nonlinear hybridization chain reaction (HCR). A multi-branched DNA product, 3DmhD, was employed to amplify target miRNA signals. The DRP comprises a gold nanocage (Au NC) core functionalized with nucleic acid probes and Raman signal molecules. SERS analysis facilitated ultrasensitive detection with high specificity in intracellular miRNA assays, suggesting DRP's potential for tumor cell screening through intracellular miRNA expression profiling. Additionally, biosensor-based approaches have been explored. Tan *et al.* 121 developed a dual-signal SERS biosensor for quantitative miR-21 detection. This biosensor exhibited high sensitivity, excellent reproducibility, superior specificity, and precise accuracy.

**Figure 12:**
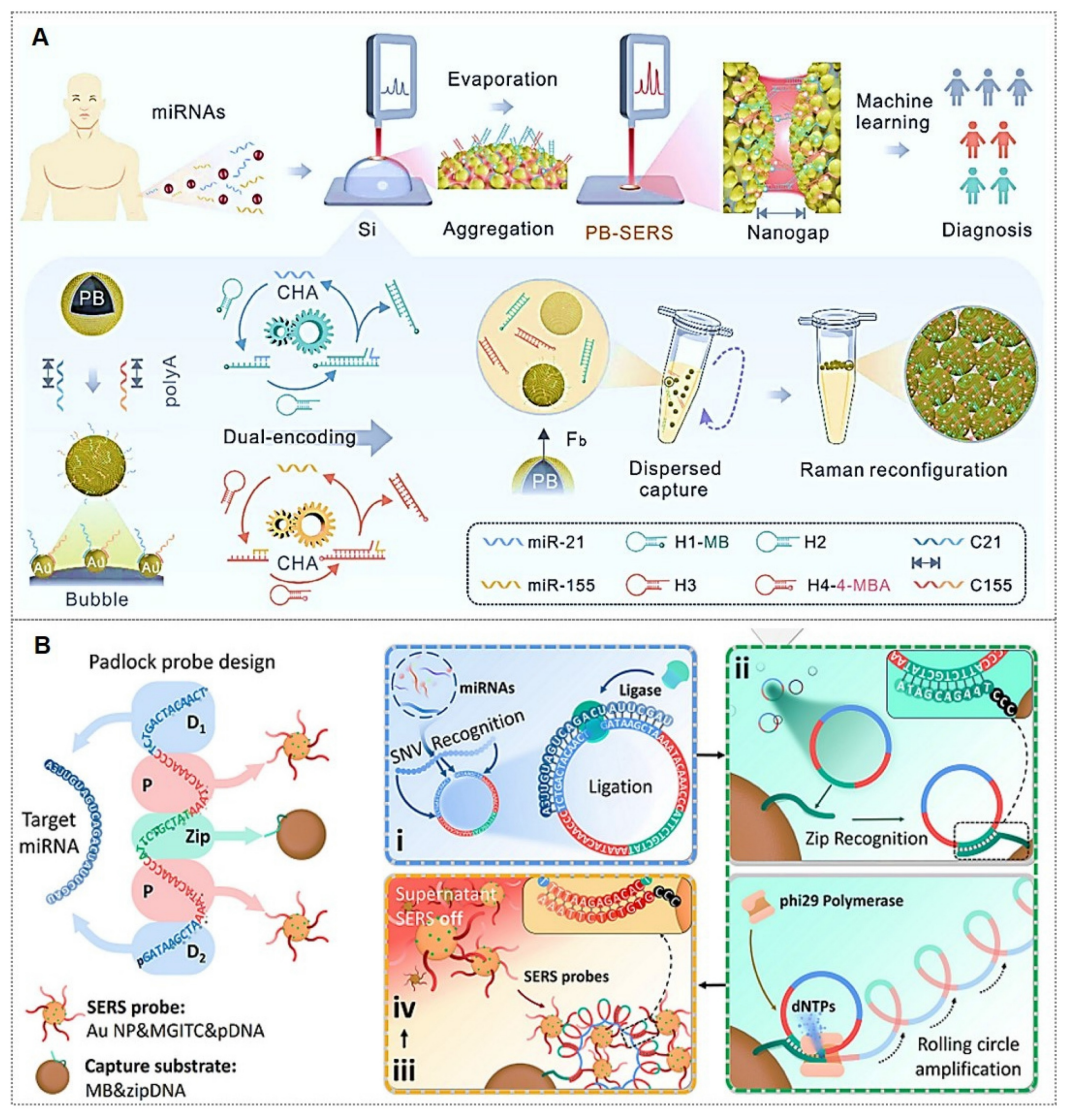
(A) PB-SERS assay for dual-miRNA detection via DNA-encoded plasmonic bubbles. Reproduced with permission from Ref. 122. Copyright 2024, Wiley. (B) Padlock probe design: Target recognition (i), RCA initiation (ii), SERS output via AuNP-RCA complexes (iii-iv). Reproduced with permission from Ref. 126. Copyright 2024, Elsevier.

Current strategies for specific binding of target miRNAs predominantly rely on complementary nucleic acid sequences. However, accurate miRNA detection remains challenging due to their low expression levels. To address these limitations, catalytic hairpin assembly (CHA) amplification strategies are frequently integrated with SERS for miRNA analysis 122-124. For instance, Yang *et al.* 122 introduced a plasmonic bubble aggregate-driven DNA-encoded SERS assay for simultaneous, specific detection of multiple miRNAs in blood, enabling precise cancer diagnosis (Figure 12A). Weng *et al.* 125 developed a SERS-CHA biosensor for detecting miRNA-21 and miRNA-155 in breast cancer serum, achieving limits of 0.398 fM and 0.215 fM, respectively, with a 1 fM-10 nM dynamic range.

Similarly, rolling circle amplification (RCA), a robust DNA amplification strategy, has been integrated with SERS labeling to construct highly sensitive miRNA detection platforms 126, 127. For instance, Qian *et al.* 126 reported a fully integrated droplet-based microfluidic platform utilizing RCA and SERS for precise detection of miRNA-21 and miRNA-155 in serum from idiopathic pulmonary fibrosis (IPF) patients (Figure 12B). The RCA-based sensor enabled single-nucleotide variant discrimination with exceptional specificity. Microfluidic integration addressed SERS reproducibility challenges, significantly improving sensitivity. Moreover, combined detection of both miRNAs demonstrated enhanced diagnostic potential, yielding an AUC value of 0.884. Wang *et al.* 127 developed an RCA and click chemistry-driven DNA hydrogel for SERS-based okadaic acid (OA) detection, integrating target-responsive DNAzyme signal amplification. The method achieved a 0.2 nmol/L LOD within 30 minutes, with SERS intensity linearly correlating to OA concentration (1.0-300.0 nmol/L), enabling rapid trace toxin detection in complex matrices.

##### 4.1.2.5. Metabolites

**Figure 13:**
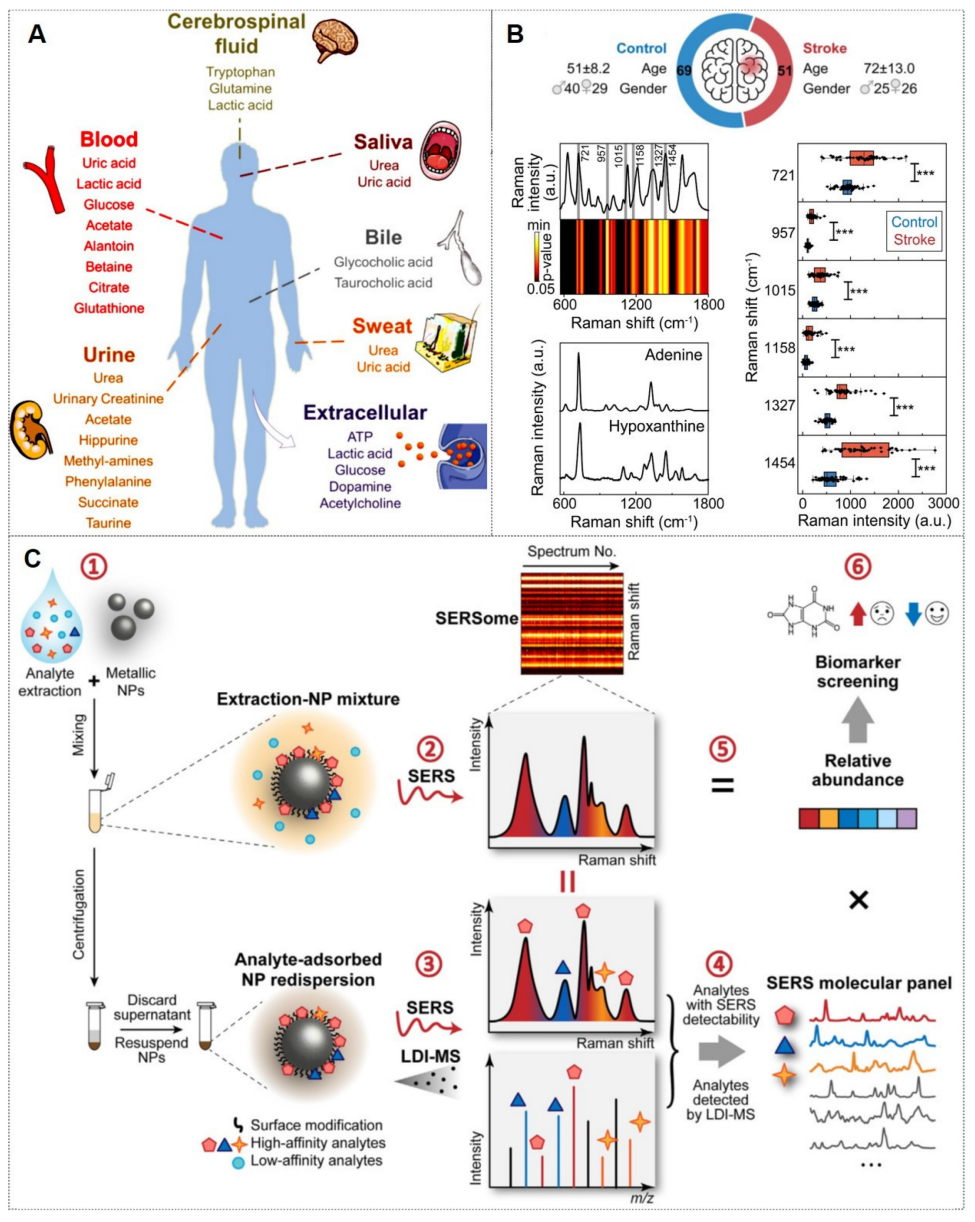
(A) Common metabolites from biofluids and cells. Reproduced with permission from Ref. 128. Copyright 2022, Elsevier. (B) A demographic profile of the healthy control group compared to stroke participants and biomarker screening for ischemic stroke. Reproduced with permission from Ref. 129. Copyright 2024, Ivyspring. (C) Workflow of MORE SERSome. Reproduced with permission from Ref. 130. Copyright 2025, Elsevier.

In recent years, metabolites have served as critical biomarkers in bodily fluids, providing direct insights into cellular metabolism and physiological states. Their analysis remains a key focus in biomedical research (Figure 13A) 128. The Ye research team has made significant contributions in this field. For instance, they identify differentially abundant metabolites from 69 controls and 51 ischemic stroke patients undergoing early reperfusion (< 24 h) via SERS and liquid chromatography-mass spectrometry (LC-MS). *In vivo* studies employed a Transient middle cerebral artery occlusion (tMCAO) mouse model with intravenous hypoxanthine administration, followed by tetrazolium chloride (TTC) staining, behavioral assessment, and Blood-brain barrier (BBB) integrity evaluation (Evans blue/IgG extravasation). Human blood vessel organoids were utilized to dissect hypoxanthine-induced endothelial pyroptosis mechanisms. SERS and LC-MS enabled high-resolution metabolic characterization of stroke serum. Hypoxanthine levels showed significant elevation in acute stroke cohorts (p < 0.001 post-Bonferroni correction). Mechanistic investigations revealed hypoxanthine triggers GSDME-mediated pyroptosis via endothelial Ca^2+^ overload in both organoid and animal models. This study identifies hypoxanthine as a pivotal metabolite driving vascular injury and BBB breakdown in stroke through hypoxanthine-mediated gasdermin E (GSDME)-dependent endothelial pyroptosis, with Ca^2+^ dysregulation as a central mechanism (Figure 13B) 129.

Moreover, multiplexed detection‌ in analytical chemistry faces significant challenges in complex systems. SERS offers a powerful solution with its molecular fingerprinting capability, high sensitivity, cost-effectiveness, and ease of use. However, spectral overlapping complicates molecular identification and quantification in ‌SERSome‌ (spectral sets) 131. They introduce ‌Molecule-Resolvable (MORE) SERSome‌, a method that identifies specific analytes within complex SERS spectra for precise spectral decomposition and multiplexed analysis. As a proof-of-concept, they applied this approach to ‌Alzheimer's disease metabolic profiling‌, successfully screening ten serum metabolites. A ‌deep-learning model‌ enabled rapid and accurate diagnosis, achieving an ‌AUC of 91.5%‌. Compared to conventional methods, ‌MORE SERSome‌ represents a methodological breakthrough in multiplexed detection, with broad applicability in analytical chemistry research and clinical diagnostics (Figure 13C) 130.

### 4.2. Bioimaging

SERS technology is not limited to sensing; it also serves as a precise imaging tool for both *in vitro* and *in vivo* specimens. Currently, SERS imaging is primarily employed for the analytical imaging of cells, tissues, and living organisms 132. Typically, SERS-based bioimaging visualizes the spatial distribution of SERS nanoprobes, whose spectral signatures reveal the molecular interactions between target analytes and plasmonic nanostructures. These interactions provide molecular-level insights into biological architectures and dynamic reaction processes. In essence, bioimaging techniques yield more intuitive and spatially resolved research data. Consequently, employing microscopic (cellular and tissue-level) or macroscopic (organ or whole-animal scale) imaging strategies to elucidate the interactions of SERS nanotags or probes with their microenvironment holds significant scientific merit. To date, SERS bioimaging technology has been extensively employed in biomedical exploration, with remarkable advancements demonstrated in related studies.

#### 4.2.1. Cellular imaging‌

**Figure 14:**
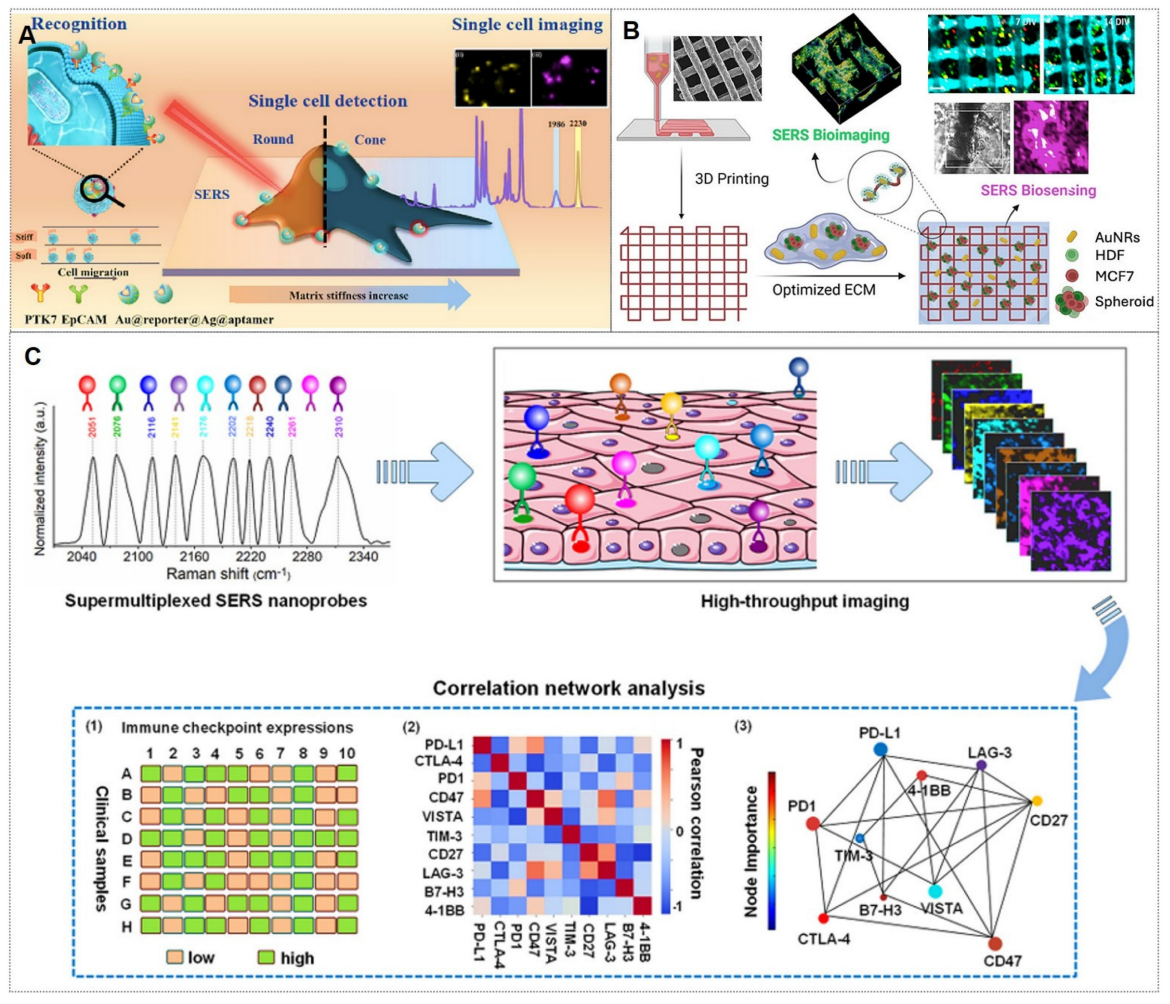
(A) Single-cell SERS imaging of dual membrane receptors on HeLa cells across substrates with tunable stiffness. Reproduced with permission from Ref. 133. Copyright 2024, Elsevier. (B) 3D cancer cell scaffold for real-time SERS sensing and imaging. Reproduced with permission from Ref. 134. Copyright 2024, American Chemical Society. (C) Multiplexed SERS nanoprobes for high-throughput imaging and correlation analysis. Reproduced with permission from Ref. 136. Copyright 2023, Elsevier.

Compared to living organisms, cells represent relatively simpler and more extensively studied biological systems. Most pathological conditions originate from subtle alterations in cellular processes, which progressively amplify over prolonged disease progression. Although numerous optical and non-optical techniques have been employed for cellular imaging analysis, SERS has rapidly advanced in this field due to its non-destructive and minimally invasive nature. For instance, Liu *et al.* 133 achieved dual-membrane receptor (PTK7/EpCAM) SERS imaging on single cancer cells using tunable PA hydrogels (2.5-25 kPa) to mimic ECM stiffness (Figure 14A). Clara Garcia-Astrain *et al.* 134 developed a 3D-printed hydrogel scaffold with plasmonic nanoparticles for SERS biosensing in 3D breast cancer spheroids (Figure 14B). Dong *et al.* 135 visualized membrane protein dimerization (e.g., Met-Met and TβRII-TβRII) on living cells, revealing cancer-stromal signaling pathways via SERS.

The narrow peak bandwidth of SERS imaging technology is widely recognized for its suitability in multiplex analysis. For example, the Li team 136 described an ultraplex SERS imaging and correlation network analysis strategy to predict synergistic immune checkpoint protein (ICP) inhibitor combinations (Figure 14C). They first constructed an ultrasensitive and ultraplex VASERS (vibrational signature-encoded Raman spectroscopy) nanoprobe platform, guided by frequency-modulation protocols to resolve 32 distinct spectral signatures. This platform enabled ten-color ICP imaging in clinical breast cancer biopsies, revealing expression heterogeneity. Correlation network analysis identified synergistic ICP combinations for immunotherapy, offering novel therapeutic perspectives.

#### 4.2.2. Tissue imaging‌

Advances in technology and instrumentation have shifted research focus beyond live-cell imaging to include comprehensive tissue imaging analysis 137. Rapid tissue imaging holds transformative potential for enhancing medical diagnostics, most notably in delineating tumor margins to guide intraoperative tumor resection. Selective intraoperative imaging is critical to ensure complete tumor removal, and SERS technology, with its capacity for rapid, sensitive, and real-time responses, presents promising prospects for tissue imaging applications.

For instance, Tian *et al.* 138 proposed a label-free SERS platform to monitor the entire process of Aβ aggregation in neurons and brain tissues in real-time. In this study, brain tissue sections from APP/PS1 transgenic mice were treated with food (control group) and metal ions, followed by incubation with the SERS platform. The nanoprobes were observed to infiltrate neurons, predominantly localizing in the hippocampal region. Raman spectral shifts at 1268 cm^-1^ and 1244 cm^-1^ revealed that Aβ aggregation was differentially modulated by metal ions. Cao *et al.* 139 achieved label-free imaging of DNA DSBs in lymphoma tissue sections (30×30 μm^2^) within 15 minutes using a SERS chip. A DCNN model analyzed clinical samples (normal vs. NHL lymphoid tissues), achieving 91.7±2.1% diagnostic accuracy. This SERS-AI strategy enables robust, minimally invasive lymphoma detection and holds potential for broader cancer diagnostics.

#### 4.2.3. *In vivo* multimodal imaging‌

SERS has emerged as a prominent focus in biomedical analysis and *in vivo* imaging due to its exceptional sensitivity, specificity, and multifunctional detection/imaging capabilities 132, 140. The information obtained by a single SERS imaging mode is of certain help for the research of complex systems. In systems combined with other imaging modes, the information obtained by other imaging modes can further verify the accuracy of SERS information and provide flexibility for the selection of multiple parameters and signals.

**Figure 15:**
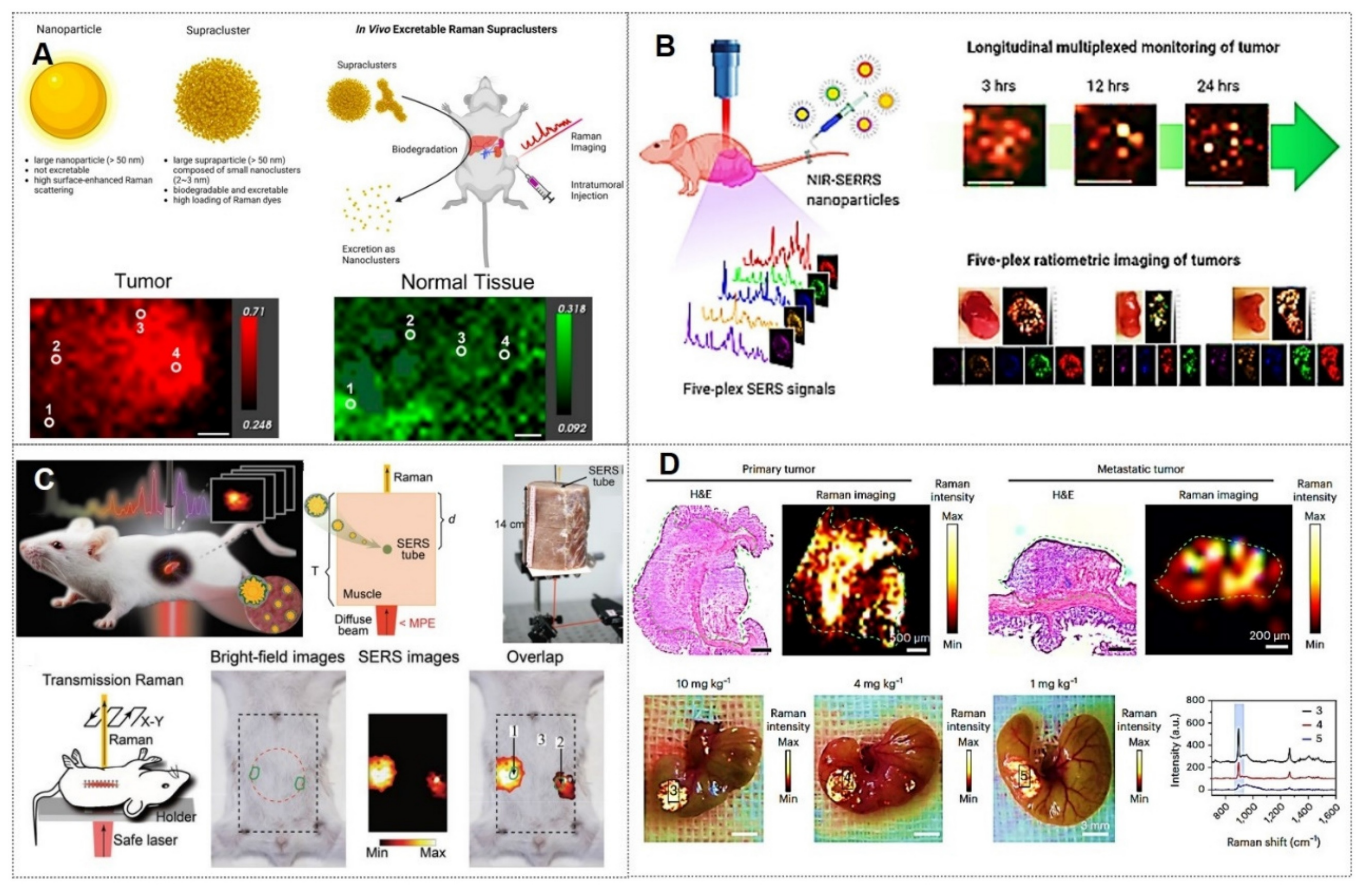
(A) Excretable Au superclusters for translational Raman imaging. Reproduced with permission from Ref. 141. Copyright 2023, American Chemical Society. (B) Five-color SERS tumor imaging with multicore nanoparticles. Reproduced with permission from Ref. 142. Copyright 2021, American Chemical Society. (C) Transmission Raman spectroscopy for deep-tumor detection. Reproduced with permission from Ref. 144. Copyright 2023, Wiley. (D) Self-stacked molecules for substrate-free Raman imaging. Reproduced with permission from Ref. 145. Copyright 2024, Nature Biotechnology.

For instance, Yu *et al.* 141 developed pH-responsive Raman superclusters for acidic tumor imaging in mice, with 73% excretion within 4 months (Figure 15A). Yu *et al.* 142 developed NIR-SERRS nanoparticle oligomers for quintuple SERS imaging in tumor-bearing mice, overcoming multiplexing limitations (Figure 15B). Shan *et al.* 143 created Au@Au-Ag cubic dotted nano-frameworks (DCFs) for NIR-II/SERS microtumor detection. Additionally, Zhang *et al.* 144 achieved 14-cm-thick transmission Raman spectroscopy (TRS) detection of SERS tags and 1.5-cm-deep *in vivo* imaging (Figure 15C). Furthermore, Gao *et al.* 145 introduced a stacking-induced charge transfer-enhanced Raman scattering (SICTERS) nanoprobes, offering 1350-fold higher scattering cross-section than Au NPs and enabling blood/lymphatic vessel imaging (Figure 15D).

### 4.3. Tumor treatment

With the advancement of SERS technology, its applications have progressively expanded from fundamental medical research to clinical practice, demonstrating the potential to emerge as a novel clinical diagnostic and therapeutic modality leveraging its unique advantages.

#### 4.3.1. Tumor boundary identification

Cancer infiltration and metastasis represent the primary factors contributing to disease progression and mortality in patients. Consequently, precise localization of tumor margins is critical during surgical resection. Currently, clinical practice relies predominantly on histopathological examination of tissue sections to confirm the presence of tumor cells in biopsy samples or employs intraoperative imaging navigation systems to delineate tumor boundaries. While histopathology remains the gold standard, it is time-consuming, requires extensive expertise, and is unsuitable for real-time intraoperative calibration. The latter approach suffers from inaccuracies due to phenomena such as “brain shift”, leading to excessive resection of healthy tissues or residual microscopic tumor foci, which may result in recurrence 77, 146.

**Figure 16:**
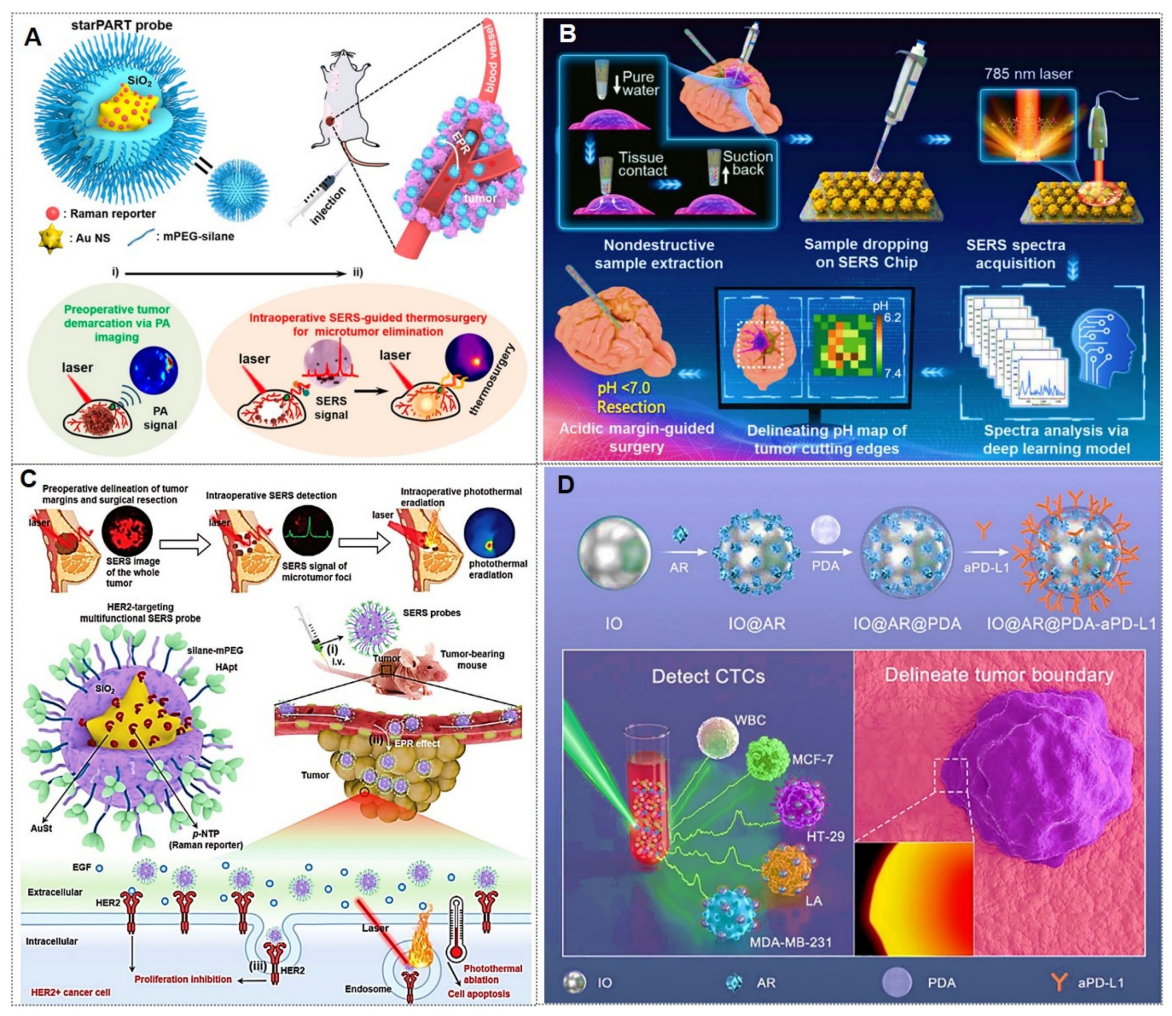
(A) StarPART probes for image-guided tumor resection and SERS-assisted thermosurgery. Reproduced with permission from Ref. 148. Copyright 2021, American Chemical Society. (B) Intelligent SERS system for brain tumor surgery via metabolic acidosis mapping. Reproduced with permission from Ref. 149. Copyright 2022, Wiley. (C) SERS-guided HER2+ breast surgery with real-time microtumor ablation. Reproduced with permission from Ref. 150. Copyright 2024, Wiley. (D) PD-L1-targeted SERS probes for CTC detection and tumor boundary delineation. Reproduced with permission from Ref. 152. Copyright 2025, Elsevier.

In recent years, SERS technology has gained prominence for its unique advantages in tumor boundary identification, thereby guiding surgical resection. The latest advancements in imaging based on nanomaterials for Raman-assisted depiction of tumor margins have demonstrated significant application potential in the fields of optics, spectroscopy, and tumor boundary depiction using hybrid nanotechnology 32, 145, 147. For instance, Li *et al.* 32 present porous nanostructures with a cubic AuAg alloy nanoshell and nanopores. These structures also show superior near-infrared II SERS performance that varies with porosity. *In vivo* experiments in a mouse model reveal their capability as SERS probes for visualizing sub-millimeter microtumors, with significant potential for precise tumor margin demarcation and microscopic tumor detection. Wen *et al.* 148 demonstrated that integrating confocal microscopy with SERS enables precise detection of tumor boundaries in murine models, overcoming limitations of preoperative photoacoustic (PA) imaging and achieving complete resection of residual tumors (Figure 16A). Jin *et al.* 149 developed a label-free intraoperative SERS navigation system for glioma acidity boundary-guided resection. Experimental validation in rat orthotopic glioma models and *ex vivo* patient tissues confirmed that acidity boundary-guided resection significantly improved overall survival rates compared to conventional methods, without requiring exogenous probes. This innovation provides a transformative prototype for the clinical translation of acidity-guided glioma surgery (Figure 16B).

In addition, Wen *et al.* 150 further reported a SERS-based surgical strategy for real-time intraoperative delineation of tumor margins and eradication of microscopic lesions, enabling complete resection of HER2+ breast tumors with no local recurrence and 100% disease-free survival. This approach holds promising potential for improving clinical outcomes in HER2+ breast cancer patients (Figure 16C). Sun *et al.* 151 conducted an in-depth SERS analysis of clinical-mimetic samples containing normal brain tissue and patient-derived glioma cells at varying concentrations using Ag NPs as substrates. Leveraging the intensity ratio of two characteristic Raman peaks (655 and 717 cm^-1^), they established a quantitative SERS strategy via artificial neural network and polynomial regression models to determine glioma cell proportions. Notably, this method accurately delineated tumor margins in freshly excised specimens, facilitating rapid and detailed intraoperative decision-making and pathological assessment. Pan *et al.* 152 developed an iron oxide (IO)-based magnetic SERS bioprobe (IO@AR@PDA-aPD-L1) functionalized with PD-L1 antibodies, enabling highly sensitive and specific detection of PD-L1-expressing CTCs in triple-negative breast cancer patients. This biocompatible probe integrates dual-modal imaging (SERS and MRI), enhancing tumor boundary differentiation for precise surgical guidance (Figure 16D).

#### 4.3.2. Tumor subtype classification‌

**Figure 17:**
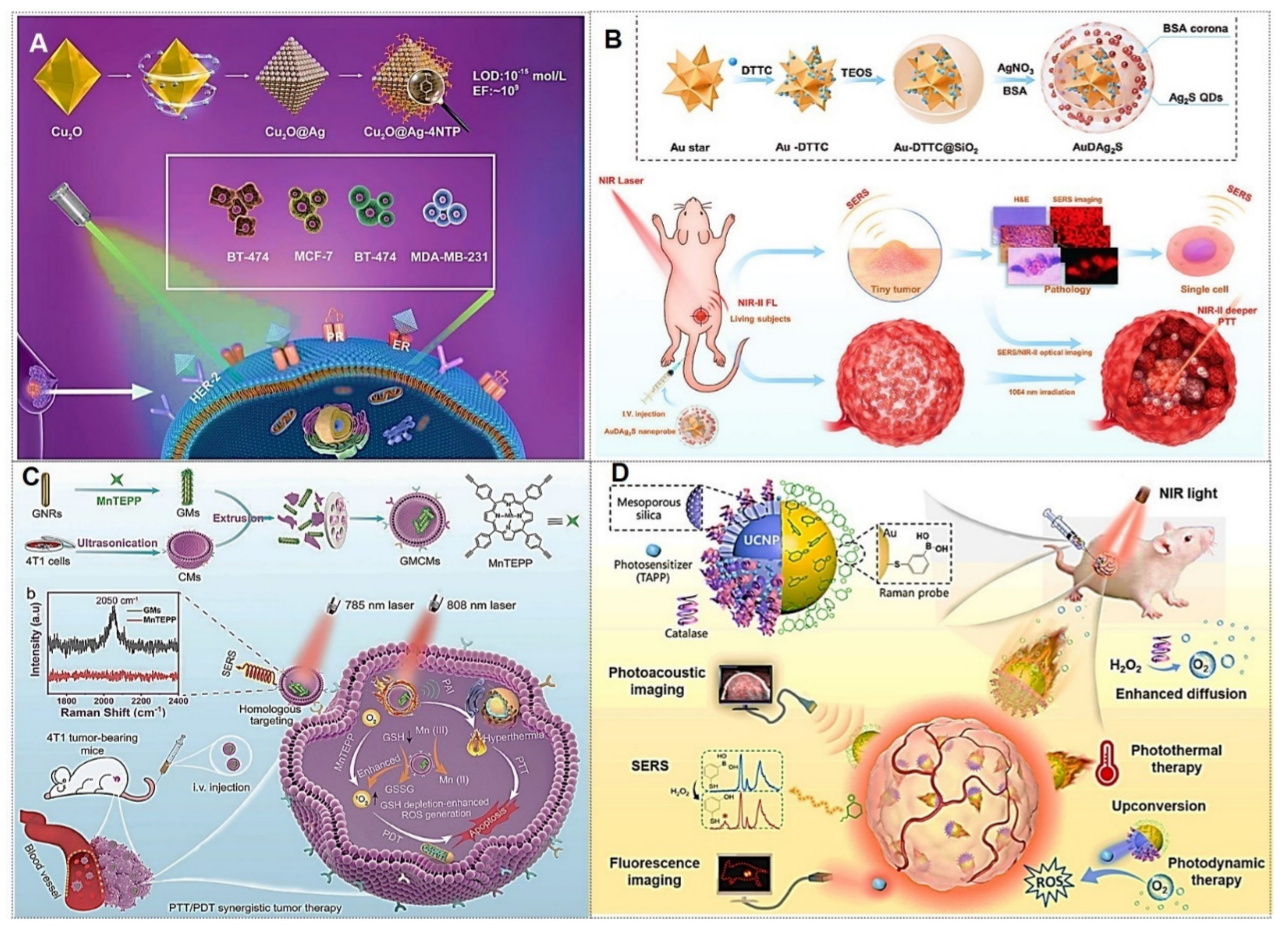
(A) Ultrahigh SERS amplification through Cu_2_O@Ag core-shell structure for breast cancer subtype classification. Reproduced with permission from Ref. 156. Copyright 2024, Elsevier. (B) AuDAg_2_S nanoprobes enabling SERS/NIR-II multimodal imaging for tumor visualization and guided photothermal therapy. Reproduced with permission from Ref. 157. Copyright 2022, Wiley. (C) Synthesis process, SERS mechanism, and PTT/PDT antitumor properties of GMCMs. Reproduced with permission from Ref. 159. Copyright 2024, Wiley. (D) Dual-powered nanomotor integrating cancer theranostic functions. Reproduced with permission from Ref. 160. Copyright 2023, Elsevier.

The classification of tumor subtypes has persisted as a formidable challenge in clinical medicine due to the striking structural similarities among distinct neoplastic subtypes, significantly impeding the rapid implementation of targeted therapies 153. The World Health Organization (WHO) classification of tumors of the central nervous system proposes substituting traditional microscopic methods with biomacromolecular profiling for brain tumor characterization. Raman spectroscopy, renowned for its capacity to interrogate molecular signatures, aligns precisely with this diagnostic paradigm. For instance, Moisoiu *et al.* 154 achieved 91% accuracy in differentiating basal and luminal subtypes of bladder carcinoma using SERS-based methodologies. Zhong *et al.* 155 employed SERS nanoprobes to label and detect matrix metalloproteinase-2 (MMP-2) within tumor subtype-specific microenvironments. By quantifying relative SERS intensities of MMP-2 across subtypes, they successfully classified breast cancer cells into normal, weakly invasive, and highly metastatic categories. Notably, Xie *et al.* 156 engineered a core-shell CuO@Ag noble metal-semiconductor heterostructure, achieving ultrahigh sensitivity and exceptional signal stability as an effective SERS probe for breast cancer subtype detection. Linear discriminant analysis of SERS spectra enabled 93.3% classification accuracy (Figure 17A).

#### 4.3.3. Multimodal tumor therapy

Recent years have witnessed continuous breakthroughs in multifunctional nanoplatforms integrating SERS and photothermal therapy for tumor theranostics. For instance, He *et al.* 157 pioneered a silica-bridged gold nanostar/Raman tag/Ag_2_S quantum dot hybrid system (AuDAg_2_S), serving as a dual-modal SERS/NIR-II all-optical probe. This platform synergized SERS molecular fingerprint imaging at picomolar sensitivity with NIR-II fluorescence imaging at 1 cm penetration depth. Under 1064 nm laser irradiation, its exceptional photothermal conversion efficiency (67.1%) enabled efficient ablation of CT26 colon cancer cells, achieving multiscale precision guidance (*in vivo*-tissue-single cell) for deep-tumor photothermal therapy (Figure 17B). This breakthrough established a prototype for all-optical visualization and treatment of deep-seated malignancies. Building on this foundation, Yin *et al.* 158 engineered a targeted theranostic platform (RGD/DOX-pAS@AuNC) by integrating mesoporous silica-coated gold nanostars with RGD-modified gold nanoclusters. This design amplified SERS “hotspots”, elevating the photothermal conversion efficiency to 85.5%. Innovatively incorporating chemotherapeutic functionality, the platform achieved a 34.1% doxorubicin loading capacity with NIR-triggered controlled release, enabling chemo-photothermal synergistic therapy. In HeLa tumor models, this system demonstrated potent tumor suppression and exceptional biosafety.

Moreover, Liu *et al.* 159 created a biomimetic nanoplatform (gold nanorod-manganese porphyrin systems, GMCMs) using 4T1 cell membranes encapsulating gold nanorod-manganese porphyrin complexes. This design enhanced targeting, dispersibility, and imaging-guided PTT/PDT efficacy via alkynyl amplification and glutathione depletion (Figure 17C). Chen's team 160 developed a dual-source-driven Janus nanomotor combining SERS sensing, fluorescence/photoacoustic imaging (PAI), photodynamic therapy (PDT), and photothermal therapy (PTT). Gold-coated nanoshells enabled PAI/PTT under 808 nm NIR, while upconversion nanoparticles (UCNPs) generated ROS for PDT, improving hypoxic tumor treatment in mouse models (Figure 17D).

### 4.4. AI-enhanced tumor diagnostics and therapy

The inherent heterogeneity, complexity, and variability of biomedical systems have long impeded molecular signature deciphering. AI-driven approaches now significantly enhance analytical precision and sensitivity, enabling robust quantification, clinical diagnostics, and fundamental biological discoveries. AI-assisted SERS spectral analysis‌ is fundamentally a process of ‌spectrum-structure relationship elucidation‌. This process typically necessitates the incorporation of various algorithms for ‌spectral-structure correlation analysis 161-169‌.

**Figure 18:**
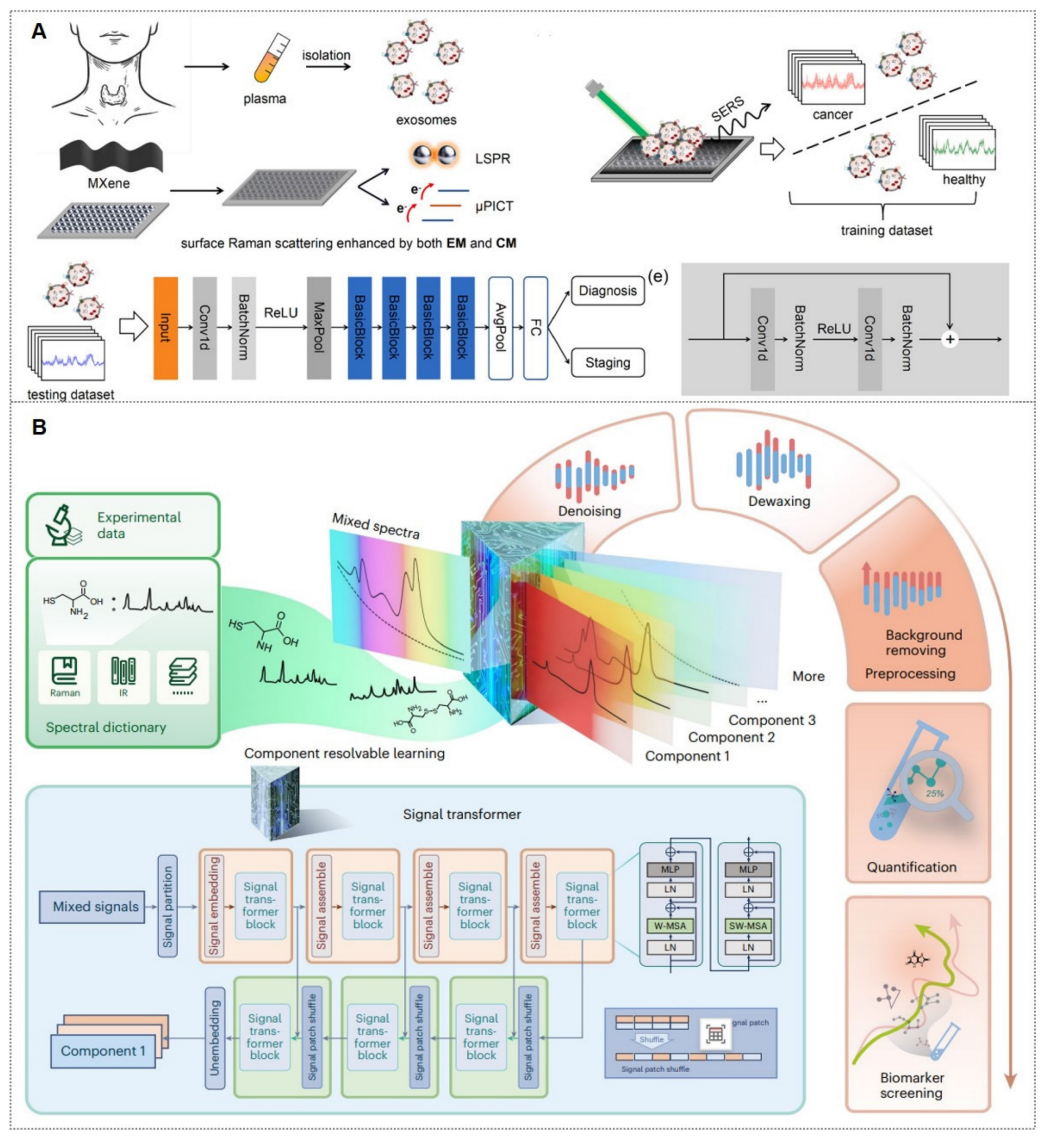
(A) Workflow: clinical sample collection, exosome isolation, MXene-coated Au@Ag NP substrate fabrication, SERS measurement, and deep learning-assisted analysis. Reproduced with permission from Ref. 161. Copyright 2024, Elsevier. (B) DSCF, as a foundation model for spectral analysis, was demonstrated in metabolic profiling. Reproduced with permission from Ref. 169. Copyright 2025, Nature Machine Intelligence.

In recent years, the introduction of machine learning algorithms such as ‌principal component analysis (PCA)‌, ‌support vector machines (SVM)‌, ‌random forests (RF)‌, and ‌convolutional neural networks (CNN)‌ has ‌significantly enhanced the sensitivity to subtle differences in SERS spectra, as well as the resolution and throughput capabilities of SERS imaging‌. For example, Sun *et al.* 161 performed label-free SERS analysis of exosomes in biological samples using MXene-coated Au@Ag NPs. A deep learning classification algorithm based on a residual neural network was subsequently developed to extract exosome-specific features from complex Raman spectra (Figure 18A). Zhao *et al.* 162 designed a g-C_3_N_4_/Ag hybrid substrate to directly collect high-intensity SERS spectra from raw exosomes derived from various cancer cells (prostate, colon, and bladder). A principal component analysis (PCA)-deep learning (DL) algorithm was constructed to analyze SERS spectra with overlapping peaks, achieving over 97.5% accuracy and 97.6% specificity in identifying cancer-specific exosomes after capturing key spectral features.

Moreover, to pursue superior sensitivity, accuracy, and analytical throughput in SERS spectroscopic analysis, it is essential to deeply integrate advanced deep learning algorithms, including diffusion models, reinforcement learning, and large language models (LLMs), thereby constructing a digital twin system for AI-enhanced SERS technology. For instance, Jian Ye's research team 169 introduces deep spectral component filtering (DSCF), a self-supervised foundation model trained via spectral component resolvable learning (Figure 18B). Acquiring domain-general spectral knowledge, DSCF achieves state-of-the-art performance across five spectral tasks on 11 datasets, demonstrating zero-shot denoising and trace quantification in complex mixtures. It enables molecule-level SERS interpretation and maps serum metabolomes from 600+ subjects for stroke, Alzheimer's, and prostate cancer studies, establishing a generalizable paradigm for spectral analysis.

Collectively, AI-assisted SERS methods have emerged as innovative tools for evaluating the spectral signatures. Perspectively, despite advances in multimodal characterization, transformative solutions remain imperative for clinical SERS translation. Critical milestones include: enhanced sensitivity/multiplexing in complex matrices, end-to-end workflow standardization, and bioethical compliance. AI-enabled implementation may catalyze the evolution from laboratory proof-of-concept to clinical deployment.

## 5. Optimization and improvement of SERS technology

Since the turn of the 21st century, advancements in SERS have prioritized advancements in substrate design (spanning novel material frameworks and engineered surface architectures), alongside the synergistic convergence of multidisciplinary methodologies. These developments tackle critical issues such as substrate uniformity, analytical reproducibility, quantification accuracy, cost efficiency, procedural simplicity, system compatibility, and long-term stability, while simultaneously broadening the functional scope of SERS applications.

### 5.1. Instrument improvement

**Figure 19:**
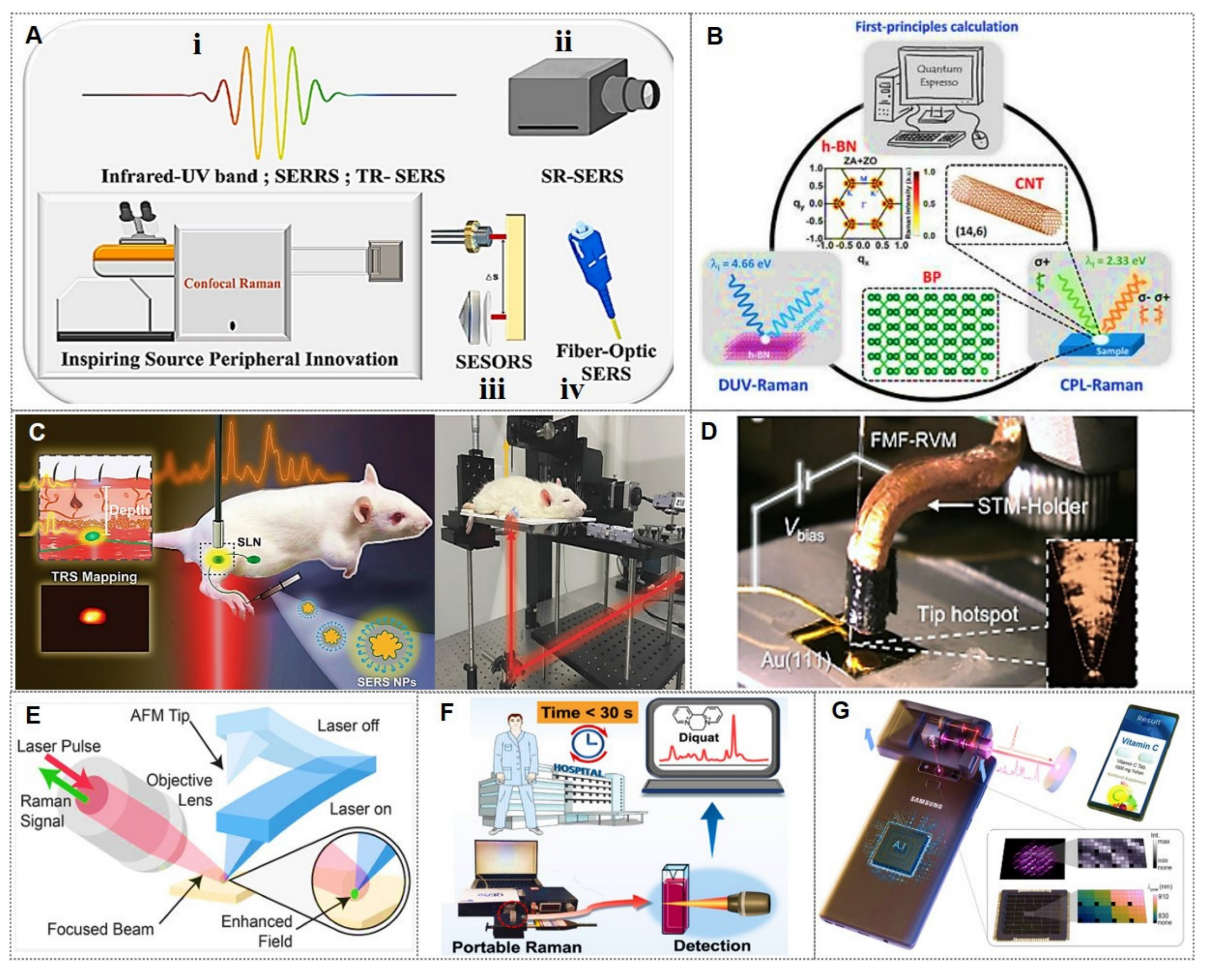
(A) SERS technology advancements: (i) Excitation sources (wavelength optimization, resonant matching, pulsed lasers); (ii) SR-SERS; (iii) SESORS; (iv) Fiber-optic SERS. Reproduced with permission from Ref. 10. Copyright 2025, Elsevier. (B) Deep-UV and helicity-dependent Raman spectroscopy for carbon nanotubes and 2D materials. Reproduced with permission from Ref. 170. Copyright 2025, Elsevier. (C) Non-invasive detection and intraoperative navigation of deep tissues using transmission Raman spectroscopy. Reproduced with permission from Ref. 171. Copyright 2023, Wiley. (D) Fiber RVM-based TERS setup with STM (inset: low-background tip hot spot). Reproduced with permission from Ref. 175. Copyright 2025, American Chemical Society. (E) Modulated-illumination intermittent-contact TERS. Reproduced with permission from Ref. 176. Copyright 2025, American Chemical Society. (F) SERS detection of DQ in biological fluids using a portable Raman spectrometer. Reproduced with permission from Ref. 177. Copyright 2023, Elsevier. (G) Smartphone Raman spectrometer with data processing workflow. Reproduced with permission from Ref. 178. Copyright 2023, Nature Communications.

The laser-analyte interplay modulates molecular polarizability, inducing Raman scattering. The incident laser's spectral properties determine both Raman emission intensity and fluorescence interference. Contemporary SERS research now explores excitation sources beyond the visible spectrum, including ultraviolet (UV) and infrared (IR) regimes (Figure 19A-i) 10, 170-172. For instance, Saito 170 studied deep ultraviolet (DUV) and helicity-dependent Raman spectroscopy of carbon nanotubes and 2D materials, revealing that first-principles calculations are crucial for understanding DUV Raman spectra and dual-resonance mechanisms (Figure 19B). Jian Ye 171, 172 examined non-invasive detection and precise localization of deep lesions using a self-made light-safe transmission Raman spectroscopy (TRS) device, offering key technical insights for TRS clinical translation (Figure 19C).

Optimizing laser wavelength selection demands rigorous evaluation of resonance parameters. Synchronization between incident/scattered photon energies and electronic transitions in target molecules induces resonance amplification, a principle enabling heightened sensitivity in surface-enhanced resonance Raman spectroscopy (SERRS) via precise resonance alignment 173. Hybridization of analytical methods unlocks complementary functionality and novel applications. For instance, time-resolved SERS (TR-SERS) combines ultrafast pulsed lasers with SERS platforms to probe femtosecond-to-picosecond molecular dynamics and plasmon-molecule interactions. Laser configuration is critical for Raman excitation, with modes tailored to specific objectives. Spatially resolved SERS (SR-SERS) enhances spatiotemporal resolution by integrating nanoscale imaging with SERS detection, enabling localized chemical characterization and structural mapping at micro/nanoscopic scales (Figure 19A-ii) 10. Synergizing spatially offset Raman spectroscopy (SORS) with SERS yields surface-enhanced SORS (SESORS), which minimizes fluorescence artifacts while improving subsurface molecular detection sensitivity (Figure 19A-iii) 10. Fiber-optic SERS probes, developed through photonic waveguide technology, offer precise optical signal modulation in constrained environments (Figure 19A-iv). These probes exhibit high electromagnetic noise immunity, mechanical flexibility, and configurable geometries, supporting real-time remote analyte tracking. Endoscopic SESORS platforms further extend SERS utility to *in vivo* diagnostics 174.

Tip-enhanced Raman spectroscopy (TERS) is pivotal for nanoscale light-matter interaction studies. However, existing TERS methods cannot simultaneously achieve low-background inhomogeneous EM field excitation and significant enhancement of electric fields, field gradients, and optical magnetic fields. To overcome this, Meng *et al.* 175 developed a TERS strategy using fiber-based vectorial optical fields to probe multipolar Raman scattering in molecules (Figure 19D). This vectorial-field-driven TERS approach enables unprecedented study of weak material responses, with applications in single-molecule spectroscopy, ultrasensitive sensing, and catalytic monitoring. Separately, G. Ruppert *et al.* 176 integrated TERS with intermittent-contact atomic force microscopy (AFM) for synchronous nanoscale mechanical imaging and chemical contrast (Figure 19E).

Portable/handheld Raman spectrometers overcome field-deployment limitations of benchtop systems for SERS applications, enabling rapid clinical diagnostics and contraband detection 177, 178. For example, Li *et al.* demonstrated 30-second diquat quantification in biological fluids for point-of-care testing (Figure 19F) 177. Smartphone-based spectroscopic innovations‌ have emerged as a transformative frontier. Separately, Un Jeong Kim *et al.*, using spectral barcodes and convolutional neural networks, achieved 99.0% accuracy in classifying 11 drug components (Figure 19G) 178. These innovations support decentralized diagnostics through integration with hyperspectral imaging and advanced algorithms.

### 5.2. SERS substrate innovation

To engineer optimized plasmonic hotspots, significant research efforts have been dedicated to investigating surface architectures and compositional configurations of substrate materials. The integration of nanoporous crystalline materials, particularly metal-organic frameworks (MOFs) and covalent organic frameworks (COFs), into SERS platforms has led to the advancement of MOF-integrated and COF-coupled SERS architectures 10. These hybrid systems effectively capitalize on electromagnetic field amplification achieved through plasmonic nanostructures embedded within the hierarchical pore networks of crystalline frameworks 179, 180. Advanced solvent-mediated nanotransfer lithography methods facilitate the fabrication of vertically aligned plasmonic nanoassemblies, producing ultra-dense and geometrically ordered 3D hotspot matrices 40. For discrete plasmonic nanoparticles, localized field enhancement predominantly occurs at topographical irregularities such as cavities, protrusions, interparticle junctions, and apex regions. Quantitative analysis reveals that gradients in surface asperity directly modulate the magnitude of Raman signal amplification 181. To improve multifunctionality, spectral tunability, and chemical robustness, noble metal cores are frequently encapsulated within functional shell materials, forming hybrid core-shell architectures 182.

For adaptable sensing applications, deformable SERS substrates employing cellulosic matrices, polymeric composites, and inorganic membranes have been engineered 183. To optimize plasmonic nanoparticle anchoring and analyte adsorption capacity, researchers have developed hierarchical 2D/3D mesoporous scaffolds and nanocellulose aerogel-based detection platforms 184. To overcome operational constraints in ultra-trace analyte localization and mitigate colloidal deposition artifacts (e.g., coffee-ring effects), superhydrophobic SERS interfaces have been engineered to achieve spatial confinement of analytes through wettability contrast strategies 185. Addressing economic feasibility, investigations have focused on eco-compatible and cost-effective natural substrates exhibiting intrinsic hydrophobicity-driven preconcentration properties 186.

### 5.3. SERS and intelligent technology integration

In recent years, the SERS-AI convergence leverages machine learning algorithms to extract chemically specific spectral features from substrate-acquired high-fidelity molecular signature data, establishing intelligent spectral deconvolution frameworks 163-166. The fundamental principle of AI-assisted SERS detection and analysis involves optimizing the signal processing, data analysis, and result interpretation of traditional SERS technology through artificial intelligence techniques. The typical workflow includes: (1) data acquisition: obtaining SERS spectral datasets; (2) preprocessing: standardizing spectral baselines and eliminating fluorescence background; (3) model training: establishing feature-label mappings using machine and deep learning algorithms; (4) result output: generating interpretable detection results, including substance identification and concentration prediction. Currently, we are entering a new era of data-driven research paradigms, where AI is accelerating the automation, high-throughput, and unsupervised preprocessing of spectral data, thereby enhancing the adaptability and transferability of SERS models.

Moreover, the confluence of SERS detection with microfluidic architectures through integrated SERS-lab-on-a-chip systems significantly improves measurement reliability and experimental repeatability under multivariable operational conditions. Microfluidic channels further enable precision engineering of SERS-active surfaces through laminar flow-controlled nanoparticle assembly and high-throughput manufacturing with spatial resolution 187, 188. These systems are increasingly recognized for their potential in enhancing reproducibility, processing efficiency, and real-time analysis through surface modification, selective capture chemistry, and microfluidic pre-concentration system components. Rapid advancements have been made in the fields of point-of-care diagnostics (POCT) 189, 190, single-cell analysis 191, 192, real-time molecular monitoring 188, 193, and microfluidic pre-concentration system components 194, 195. The following discussion highlights the forward-looking value of integrated microfluidic-SERS platforms in these domains.

**Figure 20:**
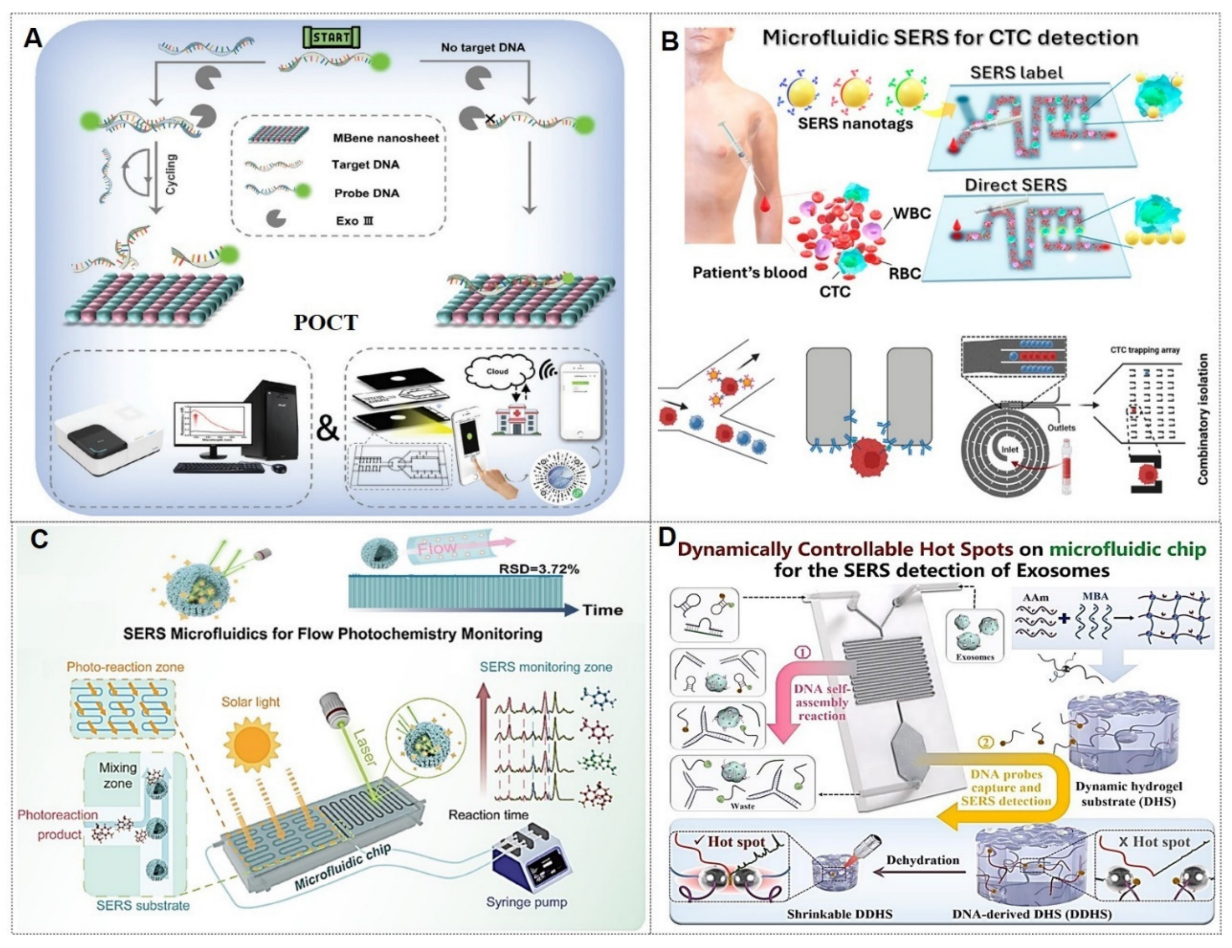
(A) MBene nanosheets with DNA adsorbability for circulating tumor DNA assay via biosensing and paper-based microfluidic POCT. Reproduced with permission from Ref. 189. Copyright 2025, Wiley-VCH GmbH. (B) SERS-microfluidic for the detection of CTCs and single-cell isolation. Reproduced with permission from Ref. 191. Copyright 2024, American Chemical Society. (C) The online SERS-microfluidics platform used for the monitoring of flow PAT photochemistry. Reproduced with permission from Ref. 193. Copyright 2025, Wiley-VCH GmbH. (D) Microfluidic-SERS chip‌: combines DDHS and DNA self-assembly for exosome detection. Reproduced with permission from Ref. 194. Copyright 2024, Elsevier.

For instance, to enable POCT ctDNA detection, the Yang group 189 developed a paper-based microfluidic chip with a delay area and mixing channel. Channel optimization via numerical simulations and experiments enhances performance. A “ctDNA detection” WeChat mini program is created for readout (Figure 20A). The Wang research group 191 has proposed a new SERS-microfluidic system, which consists of a high-throughput single-cell processing unit for separating and manipulating CTCs, and a sensitive multi-channel quantitative unit for detecting clinically relevant signals from these cells (Figure 20B). The Chen group 193 developed a colloidal SERS microfluidic platform featuring a cavity-like silver aggregate (Ag cavity. The Ag cavity demonstrates long-term stability in microfluidic channels with high reproducibility (RSD=3.72%). This work advances online chemistry studies and offers an effective tool for real-time reaction monitoring in organic synthesis (Figure 20C). Wang *et al.* 194 reported a SERS-active substrate termed DNA-derived dynamic hydrogel scaffolds (DDHS), which combines strong electromagnetic enhancement with high target affinity. Integrated into a microfluidic chip, DDHS utilized DNA oligonucleotides to recognize the exosomal marker CD63, achieving a detection limit of 2.63 particles/μL (Figure 20D).

### 5.4. SERS and multi-omics analysis methods development

**Figure 21:**
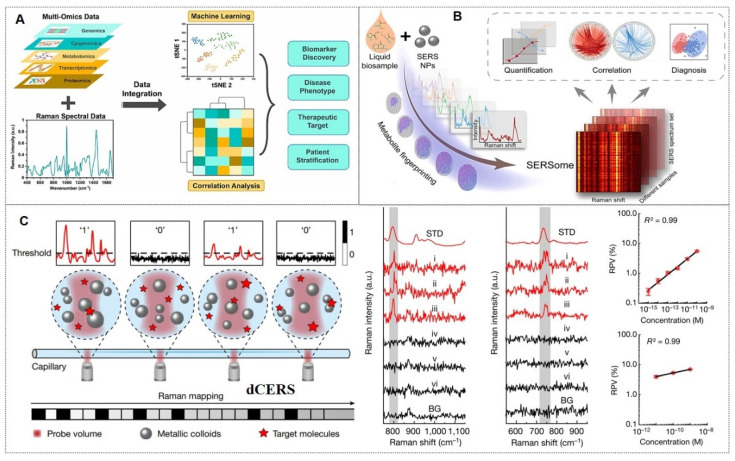
(A) Raman-omics with ML. Reproduced with permission from Ref. 197. Copyright 2023, American Chemical Society. (B) SERSomes for cancer diagnosis. Reproduced with permission from Ref. 131. Copyright 2024, Elsevier. (C) dCERS single-molecule detection. Reproduced with permission from Ref. 198. Copyright 2024, Nature.

Considering that the omics data obtained from a single Raman detection is relatively limited, researchers have begun to introduce multi-omics detection techniques to further improve the accuracy of Raman detection 196. Cutshaw *et al.* 197 emphasize the utility of SERS as an emerging omics technology for clinically relevant applications utilizing critical clinical samples and biological models. SERS enables both label-free detection of intrinsic metabolites in biomaterials and labeled tracking of *in vivo* protein biomarkers for high-throughput proteomics. Machine learning algorithms are employed to process SERS data, achieving precise therapeutic response evaluation and detection specifically targeting cancers, cardiovascular diseases, gastrointestinal disorders, and neurodegenerative pathologies (Figure 21A). Jian Ye's group has pioneered hyperspectral SERS, utilizing a spectral library termed “SERSome” to analyze biofluid metabolomes within 15 minutes 131. This approach facilitates the structural and functional characterization of diverse molecules generated under specific pathophysiological conditions in biological fluids. SERSomes present a promising methodology for metabolic phenotyping, offering insights into disease-specific metabolite signatures (Figure 21B).

### 5.5. SERS signal standardization

While SERS can theoretically detect molecular species in complex mixtures through their intrinsic vibrational fingerprints, practical implementation has been hindered by uncontrollable signal heterogeneity and poor reproducibility at low analyte concentrations. Recently, Ye's team proposed the innovative concept of digital colloidal-enhanced Raman spectroscopy (dCERS) 198, which achieves reproducible quantification of ultralow-concentration targets via single-molecule counting. Given that the metal-colloidal nanoparticles amplifying these vibrational signatures can be mass-produced under standardized conditions, dCERS is anticipated to emerge as a robust gold-standard technology for ultrasensitive detection of diverse analytes, particularly those critical to human health diagnostics (Figure 21C).

These evolutionary developments, including combinatorial derivatives, hybrid methodologies, and multimodal interfaces, fundamentally operate through plasmon-coupled Raman amplification mechanisms, systematically unified under the paradigm of plasmon-enhanced vibrational spectroscopy. This strategic integration of technological innovation with fundamental science aims to establish quantitative structure-activity relationships for nanophotonic systems while deciphering the mechanistic foundations of plasmon-mediated Raman amplification.

## ‌6. Future development and challenges

The evolution of biomedical technologies is internationally categorized into three phases: intuition-based empirical medicine, evidence-based medicine, and precision medicine governed by molecular pathology principles. Precision medicine is widely regarded as the inevitable trajectory of medical advancement. With the rising living standards and heightened public emphasis on healthcare in China, there exists an urgent imperative to advance precision medicine technologies. SERS, capable of rapidly delivering comprehensive biological insights during pre-, intra-, and post-operative phases, holds significant potential as a transformative tool for precision tumor diagnostics. Nevertheless, critical challenges persist in translating SERS into clinical oncology applications.

**(1) Standardization for clinical translation:‌** Variations in nanoscale probe preparation across different laboratories can compromise the reproducibility of results, necessitating the establishment of unified quality control standards. Key challenges to address include substrate batch-to-batch variability, standardization of detection protocols, and clinical certification. Substrate batch differences can be mitigated through material innovations, enhanced probe stability, and standardized fabrication methods. Standardization of detection workflows may be achieved via microfluidic integration, full-process automation, and operational guidelines. Furthermore, devices and technologies demonstrating significant efficacy in tumor screening and therapy should be prioritized for expedited review pathways, such as the Food and Drug Administration (FDA) fast-track approval process.

**(2) AI-assisted SERS spectral analysis:** The AI-SERS platform has exhibited exceptional performance in cancer diagnosis, treatment efficacy monitoring, and prognostic evaluation. Despite its promising potential, the translation of AI-SERS technology to large-scale clinical applications requires overcoming several hurdles. These include data standardization (requiring large-scale, multi-center, standardized clinical datasets for robust model training), model interpretability (the “black-box” decision-making mechanisms must be rendered transparent to gain clinician trust), and regulatory approval (stringent regulatory frameworks must be established to ensure safety and efficacy). In the field of spectral analysis, breakthroughs in deep learning models are redefining detection accuracy. Future research will focus on developing more stable and reliable SERS substrates, more efficient AI algorithms, and promoting large-scale clinical validation trials. In summary, this technological framework, through deep integration of algorithmic innovations and clinical needs, is advancing the transformation of spectral analysis from a laboratory tool to an intelligent medical terminal. The future integration of quantum dot-enhanced spectroscopy and lab-on-a-chip technologies holds promise for achieving a fully automated “sample-in-result-out” diagnostic closed loop.

**(3) Wearable SERS devices:**‌ As a cutting-edge technology revolutionizing biosensing and real-time detection, these systems integrate flexible substrates, microfluidic channels, and nanostructured enhancement layers to enable non-invasive monitoring of biomarkers such as sweat and interstitial fluid. Examples include wearable SERS patches (e.g., sweat-monitoring microfluidic patches) and optical tweezers for real-time dynamic tracking *in vivo*, allowing continuous observation of cellular metabolic changes, such as cancer cell responses to chemotherapeutic agents. However, current limitations include bulky device size, baseline drift due to environmental vibrations, and high per-test costs, underscoring the need for device miniaturization, signal stability improvements, and cost reduction. Future advancements may involve upgraded nanoprobes (with enhanced biocompatibility), 3D-printed nanostructures, or flexible electronics utilizing self-healing elastomeric substrates. Collectively, this technology ecosystem is shifting medical diagnostics from “centralized laboratory testing” to “personalized real-time monitoring”. Through continuous material innovations and system integration, the widespread adoption of clinical-grade wearable detection devices is anticipated in the future.

**(4) Enrichment of trace analytes in complex matrices‌:** The detection sensitivity and specificity of SERS are constrained by the inherent complexity of clinical specimens (e.g., blood, interstitial fluids) and technical limitations in isolating trace-level analytes from high-background biological matrices. At present, we can achieve this through surface modification methods, selective capture chemical reactions, and microfluidic pre-concentration systems, especially the integrated microfluidic-SERS platform for sample purification and real-time enrichment, among other emerging solutions. In the future, the convergence of microfluidics, SERS, and AI is transforming chemical and biomedical sensing. Microfluidics enables precise microscale fluid manipulation, while SERS provides ultrasensitive, label-free molecular detection. Integrating AI with microfluidic SERS enhances data processing, feature extraction, and automated decision-making, facilitating intelligent diagnostics and analysis.

In conclusion, SERS emerges as a promising modality for precise tumor surgery navigation, intraoperative detection, and personalized treatment. Future directions include integrating clinical SERS research (e.g., intraoperative identification of glioma margins, liquid biopsy diagnosis), multimodal integration (e.g., SERS-fluorescence imaging), and AI-assisted spectral analysis with standardization to further improve spectral reproducibility and advance clinical translation.

## Figures and Tables

**Table 1:** Comparison analysis of application scenarios between SERS technology and traditional common technologies.

Technology	Optimal Application Scenarios	Limited Application Scenarios
SERS 14	Trace multi-target detection	Intraoperative real-time tumor imaging, Large-scale standardized clinical testing
ELISA 15	High-throughput clinical serum screening	Early-stage diagnosis, multiplex detection
qPCR 16	Quantitative detection of pathogen nucleic acids	Non-nucleic acid targets, spatial imaging
Fluorescence imaging 17	Tracking dynamic processes in living cells	Deep tissue, long-term imaging
Mass spectrometry 18	Precision multi-omics analysis	Real-time monitoring, low-cost diagnosis

**Table 2:** The different strategies of hotspot construction for different application scenarios

Application scenarios	Hotspot construction strategies
Achieving extreme single-point enhancement (e.g., single-molecule detection) 19	Complex ‌0D nanoparticles with sharp tips/ultra-narrow gaps‌ (e.g., nanostars, nanoflowers) or meticulously engineered “hotspots within hotspots” in 3D nanostructures‌ are the preferred choices.
High reproducibility, uniformity, and quantitative analysis 20	Highly ordered arrays of 1D nanostructure gaps‌ or ‌regular arrays of 0D nanoparticles supported on 2D material substrates‌ represent the ideal options. These configurations deliver hotspots with spatially uniform distribution and stable signal output.
Solution-phase detection or biological applications 21	Dispersible 0D nanoparticles (particularly those featuring tips) or their controllable aggregates‌ are highly convenient. Their functionalization protocols are also relatively well-established.
Leverage chemical enhancement synergy 22	Selecting ‌2D materials as substrates or components within composite structures‌ (e.g., Graphene/Au NPs, MoS_2_/Ag NPs) enables the combined exploitation of EM and CM mechanisms.
Maximizing hotspot density and molecular throughput 23	Complex 3D porous or hierarchical nanostructures‌ offer the optimal solution, providing the highest density of hotspots per unit area/volume, making them suitable for high-throughput analysis or trace-level detection.
Large specific surface area to enhance molecular enrichment 24	2D nanosheets‌ and ‌3D porous structures‌ possess significant advantages.

**Table 3:** Comparative analysis of six metal nano-framework structures for SERS detection.

Structural type	Signal enhancement mechanism‌	Detection sensitivity‌	Stability‌	Application‌
WAR/WAL 35	Synergistic multi-effect (hotspots/nanopores/thermal lensing)	High	Moderate	*In situ* biological sample detection
Defect-free octahedral framework‌ 36	Electromagnetic field amplifier	Extremely high	High	Single-cell molecular tracking
‌Au triangular nanoframe with inscribed nanoring 37	Lightning rod effect	Extremely high	Outstanding	Gas-phase VOCs detection
Au octahedron-core cubic framework ‌^38^	Near-field focusing	Ultra-sensitive	High	Trace toxin screening
Pt@Ag/Pt@Au heterostructure ‌^39^	Plasmonic coupling	High	Moderate	Complex pollutant matrix analysis
Au dual-wall nanoframe ‌^40^	Sub-nanogap localization	Extremely high	Moderate	Gas-phase trace detection

**Table 4:** Typical Raman reporters and their characteristic peaks.

Spectral regimes	Raman reporter	Characteristic peak (cm^-1^)	Reference
Fingerprint region	Malachite green	1614	49
	Nile blue	591	49
	Astra blue	1539	49
	4-mercaptobenzoic acid	1586	54
	4-acetamidothiophenol	1073	55
	4-aminothiophenol	1079	56
	1, 2-bis(4-pyridyl)ethylene	1612	57
	2-mercaptohydroquinone	985	58
	Rhodamine B isothiocyanate	1643	59
	5,5'-dithio-bis-nitrobenzoic acid	1334	60
	2-thienyl-substituted chalcogenopyrylium	1600	50
	Cyanine7-Styramide	586	51
Cellular silent region	4-mercaptobenzonitrile	2227	61
	Sucrose analogues (alkyne)	2116	62
	Bisarylbutadiyne	2213	63
	Sodium thiocyanate	2115	64
	KxMFe(CN)_6_, (M=Pb, Ni)	2139, 2197	65
	Metal carbonyls (M(CO)_6_, (M=Re, W, Mo)	2021, 2067, 2070	66

**Table 5:** Response ranges and application scenarios of environmentally responsive molecules.

Type	Responsive molecule	Response range	Application scenarios	Ref
pH	4-MBA	4.5~6.0	Tumor precision diagnosis	90
	4-MPY	7.35~7.45	Rapid identification of glioma boundaries	91
	BCCDP	3.0~9.0	Biological detection, bioimaging, and environmental monitoring	92
	4-MPBA	5.0~7.4	Cancer cell targeting, pH-sensitive drug release, and SERS-MR multimodal tracking	81
Temperature	PNIPAAm	37°C	Implantable SERS-active polymeric meshes with thermoresponsive properties	93
Redox	Resazurin	/	Quantifying antioxidant capacity in biological systems	94
	2-MBQ	/	Visualizing redox dynamics in live cells	95
	HQ	-200∼100 mV	Real-time monitoring of drug-induced oxidative stress and hypoxia	96
Light	DTTC	/	Depth-resolved tumor imaging and real-time drug release monitoring via plasmonic tags	97
